# Genome-wide analysis of the plant-specific PLATZ proteins in maize and identification of their general role in interaction with RNA polymerase III complex

**DOI:** 10.1186/s12870-018-1443-x

**Published:** 2018-10-05

**Authors:** Jiechen Wang, Chen Ji, Qi Li, Yong Zhou, Yongrui Wu

**Affiliations:** 1 National Key Laboratory of Plant Molecular Genetics, CAS Center for Excellence in Molecular Plant Sciences, Institute of Plant Physiology & Ecology, Shanghai Institutes for Biological Sciences, Chinese Academy of Sciences, 300 Fenglin Road, 200032 Shanghai, People’s Republic of China; 2 University of the Chinese Academy of Sciences, Beijing, 100049 China

**Keywords:** Maize, Transcription factor, PLATZ, RNA polymerase III, RPC53, TFC1

## Abstract

**Background:**

PLATZ proteins are a novel class of plant-specific zinc-dependent DNA-binding proteins that are classified as transcription factors (TFs). However, their common biochemical features and functions are poorly understood.

**Result:**

Here, we identified and cloned 17 *PLATZ* genes in the maize (*Zea mays*) genome. All ZmPLATZs were located in nuclei, consistent with their predicted role as TFs. However, none of ZmPLATZs was found to have intrinsic activation properties in yeast. Our recent work shows that FL3 (ZmPLATZ12) interacts with RPC53 and TFC1, two critical factors in the RNA polymerase III (RNAPIII) transcription complex. Using the yeast two-hybrid assay, we determined that seven other PLATZs interacted with both RPC53 and TFC1, whereas three had no protein-protein interaction with these two factors. The other six PLATZs interacted with either RPC53 or TFC1. These findings indicate that ZmPLATZ proteins are generally involved in the modulation of RNAPIII-mediated small non-coding RNA transcription. We also identified all of the PLATZ members in rice (*Oryza sativa*) and *Arabidopsis thaliana* and constructed a Maximum likelihood phylogenetic tree for ZmPLATZs. The resulting tree included 44 members and 5 subfamilies.

**Conclusions:**

This study provides insight into understanding of the phylogenetic relationship, protein structure, expression pattern and cellular localization of PLATZs in maize. We identified nine and thirteen ZmPLATZs that have protein-protein interaction with RPC53 and TFC1 in the current study, respectively. Overall, the characterization and functional analysis of the PLATZ family in maize will pave the way to understanding RNAPIII-mediated regulation in plant development.

**Electronic supplementary material:**

The online version of this article (10.1186/s12870-018-1443-x) contains supplementary material, which is available to authorized users.

## Background

In plants, 84 putatively TF families and other transcriptional regulators (TRs) have been identified from 19 species whose genomes have been completely sequenced and annotated (Plant Transcription Factor Database, PlantTFDB3.0) [1]. TFs are proteins that bind to cis-elements in their target promoters in a sequence-specific manner, whereas TRs exert their regulatory function through protein–protein interactions or chromatin remodelling [2].

Plants and animals or yeast do not show a good corresponding relationship in the evolution of the TF families. Approximately 50% of TFs in *Arabidopsis* and 45% in maize are plant-specific, indicating that these TFs play important roles in processes specific to plants, including secondary metabolism, responses to plant hormones, and the identity of specific cell types [3, 4]. Additionally, several TF families such as MYB superfamily, bHLH, and bZIP are large families in plants [5–7], but their numbers are remarkably fewer in animals and yeast [8, 9].

The PLATZ TF family is a novel class of plant-specific zinc-dependent DNA-binding proteins. The first reported member was PLATZ1, which was isolated from pea (*Pisum sativum*) [10] and shown to bind nonspecifically to A/T-rich sequences and repress transcription. However, the mutants and biological functions of any member in this family were not identified until the maize *Fl3* gene was cloned from a classic endosperm semi-dominant mutant. *Fl3* encodes a PLATZ protein that interacts with the RNAPIII subunits RPC53 and TFC1 through which it regulates the transcription of many transfer RNAs (tRNAs) and 5S ribosomal RNA (5S rRNA), and as a consequence, maize endosperm development and filling [11].

RNAPIII is the largest enzyme complex among RNA polymerases, which is composed of 17 subunits and is responsible for the synthesis of a range of short noncoding RNAs (ncRNAs), including 5S rRNA, U6 small nuclear RNA (U6 snRNA), and different tRNAs, many of which have functions related to ribosome and protein synthesis [12, 13]. The high energetic cost of synthesizing these ncRNAs by RNAPIII is thought to underlie an accurate and coordinated regulation to balance cell survival and reproduction.

In yeast, the RNAPIII transcription complex requires three transcription factors in addition to Pol III: two general transcription factors, TFIIIB and TFIIIC, and a specific transcription factor, TFIIIA, which is only required for the synthesis of 5S rRNA [14]. Maf1 is a master regulator in the RNAPIII transcription system in yeast, which is essential for modulating transcription under changing nutritional, environmental and cellular stress conditions [15, 16]. Nhp6 is another small but powerful effector of chromatin structure in yeast, with a function involved in promoting RNAPIII transcription at a high temperature [17].

Despite these findings in yeast, the components and mechanisms that modulate RNAPIII transcription in plants are little understood. CsMAF1 from *Citrus sinensis* was the first characterized RNAPIII-interacting protein in plants, which can interact with the human RNAPIII and repress tRNA^His^ synthesis in yeast [18, 19], indicating that the functions of MAF1 proteins are evolutionally conserved across different kingdoms. Another example is UBL1, a putative RNA exonuclease belonging to the 2H phosphodiesterase superfamily, which possesses RNA exonuclease activity in vitro and is involved in biogenesis of snRNA U6. The structure and function of UBL1 is conserved in plants, human and yeast, although the plant UBL1 is only 25.8% and 20.6% identical to its human and yeast counterpart, respectively [20].

Grain filling in maize and other grasses is a high energy-cost process for the synthesis and accumulation of starch and storage proteins, which require an accurate and coordinated regulation of ribosome and protein synthesis. FL3 (ZmPLATZ12) is specifically expressed in maize endosperm starchy cells and functions as a modulator of the RNAPIII transcription complex consistent with the highly abundant synthesis of tRNAs and 5S rRNA in the maize endosperm. Genome-wide identification and characterization of PLATZs and analysis of their interaction with RNAPIII in maize will provide an avenue for understanding the common and specific features of each PLATZ member in plant development.

## Methods

### Plant growth conditions

The maize inbred line A619 seeds were originally obtained from the Maize Genetics Cooperation Stock Center (accession number 3405–001) and planted at our institute farm in Shanghai in the summer of 2017. Tobacco (*Nicotiana benthamiana*) plants were grown in a growth chamber under a day/night regime of 16/8 h at a temperature of 20–25 °C.

### Database search and sequence retrieval

First, the maize PLATZ proteins were used to search against the PlantTFDB (http://plntfdb.bio.uni-potsdam.de/) and GrassTFDB (http://www.grassius.org/grasstfdb.php) databases. Second, the FL3 (ZmPLATZ12) protein sequence was used as a query to search against National Center for Biotechnology Information (NCBI) using the BLASTP program in the maize B73 genome version 4 (E-value ≤ e-05). The unique sequences from the three databases were used for this study. Third, the FL3 (ZmPLATZ12) protein sequence was used as a query to search against NCBI using the BLASTP program in *Oryza sativa* (japonica cultivar-group, taxid: 39947) (E-value ≤8e-18) and *Arabidopsis thaliana* (taxid: 3702) (E-value ≤2e-05) reference protein databases. Fourth, the identified rice and *Arabidopsis* PLATZ proteins (OsPLATZs and AtPLATZs, respectively) from the above were used to search against the PlantTFDB database. The unique sequences from the two databases were used for this study.

### RNA preparation, reverse transcription-PCR (RT-PCR) and cloning of PLATZ genes

Tissues (root, stem, the third leaf and SAM) were collected from at least three healthy plants at 32 days after sowing. The tassel 1, tassel 5 and ear were sampled as described previously [21]. Developing kernels were harvested at 1, 3, 6, 8, 10, 12, 14, 18, 24, and 30 days after pollination. Total RNA from fresh tissues was extracted using TRIzol reagent (Invitrogen, USA) and then purified with an RNeasy Mini Kit (Qiagen, Germany). The first-strand cDNAs were synthesized using SuperScript III reverse transcriptase (Invitrogen, USA) following manual instructions. The full open-reading frame of each *ZmPLATZ* gene was amplified with a specific primer pair. All primers used for RT-PCR are listed in Additional file 1: Table S1. The maize GRMZM2G105019 was used as the reference [22]. Fifteen *ZmPLATZ* cDNAs were amplified from the leaf, stem, tassel, endosperm or embryo tissue, with the exceptions *ZmPLATZ1* and *ZmPLATZ8*. The coding sequences of *PLATZ1* and *8* were synthesized at Sangon Biotech (Shanghai, China) Co., Ltd., based on the gene annotation.

### Expression patterns of *PLATZ* genes in B73

Expression patterns of fifteen maize *PLATZ* genes were summarized based on the maize reference genome B73 (Additional file 2: File S1) [21]. Hierarchical clustering of fifteen genes and heat map of 53 different seed samples were carried out by using normalized gene expression values with log2 (RPKM + 1) in R package ‘pheatmap’. Fifty-three samples represent different tissues and different developmental stages of the whole seed, endosperm and embryo.The sample IDs were used as previously described [21].

### Structure and phylogenetic analysis

The amino acid sequences translated from the ZmPLATZ CDSs were used to predict conserved domains using the Pfam database of Hidden Markov Model with an i-value threshold at 1.0 (http://pfam.sanger.ac.uk/search) [23] and SMART database of default parameters (http://smart.embl-heidelberg.de/) [24]. The complete amino acid sequences of ZmPLATZs, were submitted to the Clustal W program using the default settings (pairwise alignment options: gap opening penalty 10, gap extension penalty 0.1; multiple alignment options: gap opening penalty 10, gap extension penalty 0.2, gap distance 4, no end gaps and protein weight matrix using Gonnet) for for multiple protein alignment. Based on the aligned protein sequences, the ZmPLATZ phylogenetic tree was constructed using the MEGA7.0 program (http://www.megasoftware.net/) and the maximum likelihood method with Jones-Taylor-Thornton (JTT) Model, and the bootstrap test was conducted with 1000 replicates. The amino acid sequences of ZmPLATZs, OsPLATZs and AtPLATZs were submitted to the Clustal W program using the default settings for multiple protein alignment. Based on the aligned protein sequences, sequences with > 30% gap was removed. Then, a maximum likelihood tree about ZmPLATZs, OsPLATZs and AtPLATZs was constructed using the default settings based on Jones-Taylor-Thornton (JTT) Model with partial deletion and 70% Site Coverage Cut off, and the bootstrap test was conducted with 1000 replicates.

### Subcellular localization of PLATZ proteins

The amino acid sequences translated from the ZmPLATZ CDSs were used to predict nuclear localization signal (NLS) using the wolf-psort (https://psort.hgc.jp/) or PredictNLS (https://rostlab.org/owiki/index.php/ PredictNLS) online tool. The C-terminal of each *ZmPLATZ* CDS was fused to a reporter gene encoding the enhanced GFP (eGFP), which was then cloned into pCAMBIA1301 plasmid driven by the 35S promoter. *Agrobacterium tumefaciens* (strain GV3101) harbouring this construct was infiltrated into 3-week-old *N. benthamiana* leaves using a needle-less syringe. At least three replicates were performed. The eGFP signal was observed and imaged using a confocal microscope (FV1000, Olympus, Japan).

### Yeast two-hybrid assay

Full-length coding sequences of *PLATZs* were cloned into the pGBKT7 plasmid (BD) and transformed into yeast strain Y2HGold to test for auto-activation. Yeast on SD/−Trp agar plates were grown at 28 °C for 2 days and on SD/−Trp -Ade -His for 3 days. For the protein-protein interaction assay, TFC1 and RPC53 were ligated to the pGADT7 plasmid (AD). pGADT7-TFC1 or pGADT7-RPC53 with pGBKT7-PLATZs were co-transformed into Y2HGold. The yeast cells were plated on SD/−Trp -Leu at 28 °C for 2 days and on SD/−Trp -Leu -Ade -His for 3 days.

## Results

### Identification of ZmPLATZs in the maize genome

To characterize the number of members in this new family, we searched the maize PLATZ proteins in the PlantTFDB and GrassTFDB databases, which were both based on the B73 genome version 3. This search resulted in the identification of 21 and 15 members from the two databases. Although 26 completely unique protein sequences were characterized, only 15 *PLATZs* were confirmed as expressed genes by the public maize RNA-seq data [21]. Because the B73 genome version 4 is available now [25], BLASTP searches were performed using the FL3 (ZmPLATZ12) protein sequence with E-value ≤ e-05. Fourteen ZmPLATZs from version 3 were re-identified in the version 4 genome, with PLATZ2 exception, whereas two new *PLATZ* genes (Zm00001d046688 and Zm00001d046958) missing in version 3 were annotated in version 4. Collectively, 17 ZmPLATZ members including the previously reported FL3 (ZmPLATZ12) [11] were analysed in the current study (Table 1). The protein nomenclature was in accordance with that of the GrassTFDB ID (ZmPLATZ1–15), and the two new PLATZs annotated from version 4 were designated ZmPLATZ16 and ZmPLATZ17 (Table 1). The 17 *ZmPLATZ* genes are unevenly distributed on 7 chromosomes, with chromosomes 1, 5 and 9 each bearing 4 members.

**Table 1:** 17 ZmPLATZs identified from the completed maize genome sequence

Family members	Model V3	Model V4	Chromosome No.^a^	Chromosome	Position^a^	Chromosome Strand^a^
				From	To	
ZmPLATZ1	GRMZM2G408887	Zm00001d028594	1	40221978	40223031	+
ZmPLATZ2	GRMZM2G311656	Zm00001d029437	1	70442356	70448204	+
ZmPLATZ3	GRMZM2G094168	Zm00001d030032	1	101087719	101089688	+
ZmPLATZ4	GRMZM2G171934	Zm00001d031925	1	206932526	206934498	+
ZmPLATZ5	GRMZM2G131280	Zm00001d002489	2	13850163	13852195	+
ZmPLATZ6	GRMZM2G342691	Zm00001d051376	4	156414419	156415562	–
ZmPLATZ7	GRMZM2G091044	Zm00001d051511	4	160984327	160986154	–
ZmPLATZ8	GRMZM2G017882	Zm00001d015394	5	88146164	88150303	+
ZmPLATZ9	GRMZM2G070295	Zm00001d015560	5	97682870	97684322	+
ZmPLATZ10	GRMZM2G323553	Zm00001d015868	5	127438923	127439977	–
ZmPLATZ11	GRMZM2G004548	Zm00001d017682	5	204120837	204122561	–
ZmPLATZ12 (Fl3)	GRMZM2G006585	Zm00001d009292	8	52707946	52709109	–
ZmPLATZ13	GRMZM2G093270	Zm00001d047025	9	115708968	115711076	+
ZmPLATZ14	GRMZM2G077495	Zm00001d047250	9	124047961	124049024	+
ZmPLATZ15	GRMZM2G086403	Zm00001d026047	10	136891569	136893726	–
ZmPLATZ16		Zm00001d046688	9	102440929	102445873	–
ZmPLATZ17		Zm00001d046958	9	113035703	113040261	–

^a^The gene position in chromosome was according *Zea mays* B73 genome sequence Vision4

### Cloning and domain prediction of ZmPLATZs

RT-PCR was employed to amplify the intact CDS of each *ZmPLATZ* gene. PLATZ2, 5, 7, 11, 12, and 13 were cloned from the 12-DAP endosperm, and PLATZ3, 16, and 17 were cloned from the root. PLATZ4, 6, 9, 10, 11, 14, and 15 were cloned from the 18-DAP endosperm, tassel, 20-DAP embryo, 6-DAP endosperm, 12-DAP endosperm, 3-DAP seed, and 36-DAP endosperm, respectively. The expression of PLATZ1 and 8 was not detected in any tissue used in this study. (Additional file 2: File S1). The cDNA sequences of *ZmPLATZ2*, *ZmPLATZ3*, *ZmPLATZ5*, *ZmPLATZ7*, *ZmPLATZ10*, *ZmPLATZ13* and *ZmPLATZ15* were identical to the predicted CDSs from the B73 genome version 3, whereas those of *ZmPLATZ4*, *ZmPLATZ9*, *ZmPLATZ11* and *ZmPLATZ14* had several mismatches compared with the predicted CDSs (Additional file 3: Figure S1). The version 3 predicted *ZmPLATZ6* CDS was different from that of version 4 at the C-terminal. We sequenced the amplified *ZmPLATZ6* cDNA, which was nearly identical to the version 4 CDS except for 9 SNPs (Additional file 4: Figure S2). The cloned cDNA sequences of *ZmPLATZ16* and *ZmPLATZ17* were the same as the predicted CDSs of version 4 except for a 3-bp insertion in the *ZmPLATZ17* cDNA.

**Fig. 1:**
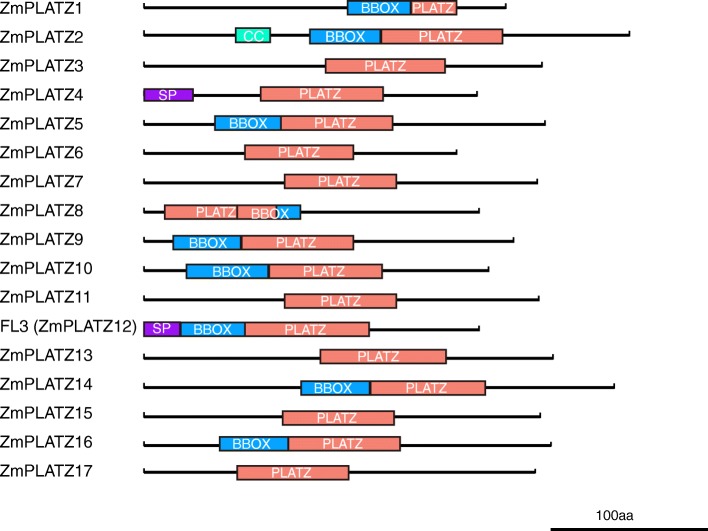
Schematic diagram of ZmPLATZs. The putative domains or motifs were identified using the Pfam and SMART databases with the default parameters. PLATZ, PLATZ domain; BBOX, B-Box-type zinc finger; SP, signal peptide; CC, coiled coil. Bar, 100 aa

PLATZ proteins were classified as TFs containing a conserved PLATZ domain, although the components of other domains have not been recognized. The protein sequences of 15 cloned and 2 predicted (*ZmPLATZ1* and *ZmPLATZ8*) *ZmPLATZ* genes were subject to conserved domains prediction using the Pfam [23] and SMART [24] databases. It was predicted that all ZmPLATZ members contained a PLATZ domain (Pfam family PLATZ: PF04640, http://pfam.xfam.org/family/PLATZ). Additionally, many members were predicted to bear a BBOX(B-Box-type zinc finger, SMART accession number: SM00336, http://smart.embl-heidelberg.de/smart/do_annotation.pl?ACC=SM000336&BLAST=DUMMY)domain, which is located before the PLATZ domain. The PLATZ domain is highly conserved between ZmPLATZs which could be identified though all the database and the BBOX domain is not very conserved with highly E-value. ZmPLATZ8 was an exception, with the BBOX positioned in the rear of the PLATZ domain with an overlap (Fig. 1, Table 2 and Additional file 5: File S2). Only ZmPLATZ2 has a CC (coiled coil) domain, and ZmPLATZ4 and ZmPLATZ12 have a signal peptide domain.

**Table 2:** Identification protein domains of 17 PLATZs by Pfam and SMART databases

Family members	Model V4	CDS Length	Signal Peptide	PLATZ Domain	BBOX Domain	Coiled-Coil	Low Comlexity Region	
ZmPLATZ1	Zm00001d028594	231		170–199	129–169		36–53	
ZmPLATZ2	Zm00001d029437	309		155–229	111–154	68–89	269–291	
ZmPLATZ3	Zm00001d030032	254		109–185			21–34,79-97,205–216	
ZmPLATZ4	Zm00001d031925	212	1–31	74–152			160–178	
ZmPLATZ5	Zm00001d002489	256		87–158	46–86		14–27,192-216,220–234	
ZmPLATZ6	Zm00001d051376	198		64–134			168–181	
ZmPLATZ7	Zm00001d051511	251		89–160			15–28,63-78,170-182,197–230	
ZmPLATZ8	Zm00001d015394	214		13–84	59–97		102–110,173–185	
ZmPLATZ9	Zm00001d015560	237		62–134	19–62			
ZmPLATZ10	Zm00001d015868	220		80–152	27–79		173–188	
ZmPLATZ11	Zm00001d017682	251		88–159			8–27,169-183,194–230	
ZmPLATZ12 (Fl3)	Zm00001d009292	214	1–24	65–144	25–64			
ZmPLATZ13	Zm00001d047025	261		112–192			21–36,82-92,232–242	
ZmPLATZ14	Zm00001d047250	299		143–217	99–142		5–10,19-43,57-70,73-88,244-256,268–277	
ZmPLATZ15	Zm00001d026047	253		88–159			15–26,170-189,195–213	
ZmPLATZ16	Zm00001d046688	259		92–163	46–91		183–195	
ZmPLATZ17	Zm00001d046958	250		59–129			188–203	

### Phylogenetic analysis of ZmPLATZs

**Fig. 2:**
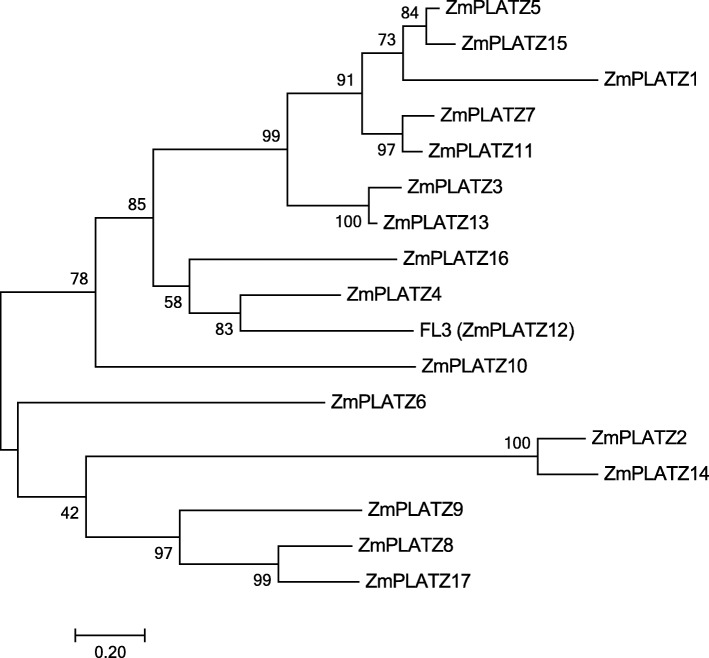
Phylogenetic analysis of ZmPLATZs. Maximum likelihood phylogenetic tree summarizes the evolutionary relationships among ZmPLATZs. The numbers under the branches refer to the bootstrap value of the maximum likelihood phylogenetic tree. The length of the branches is proportional to the amino acid variation rates

To characterize the phylogenetic relationships among ZmPLATZ proteins, we constructed a phylogenetic tree of the 17 ZmPLATZs (15 cloned and 2 predicted (*ZmPLATZ1* and *ZmPLATZ8*)) using Clustal W and MEGA 7.0. The maximum likelihood method was used to construct the phylogenetic tree (Fig. 2 and Additional file 6: Figure S3). The ZmPLATZs were grouped into three branches. Clade 1 contained ZmPLATZ5, ZmPLATZ15, ZmPLATZ1, ZmPLATZ7, ZmPLATZ11, ZmPLATZ3, andZmPLATZ13. Clade 1 ZmPLATZ members contained a conserved domain (MAID-x_4–8_-L-x_4_-R-x_4–5_-GGG) in N-terminal (Additional file 6: Figure S3). Clade 2 contained ZmPLATZ16, ZmPLATZ4, ZmPLATZ12, and ZmPLATZ10. Clade 3 contained ZmPLATZ6, ZmPLATZ2, ZmPLATZ14, ZmPLATZ9, ZmPLATZ8, and ZmPLATZ17.

### Spatial and temporal expression patterns of *ZmPLATZs*

**Fig. 3:**
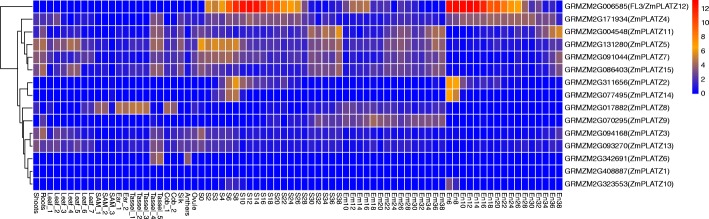
Expression patterns of the ZmPLATZ genes analysed by the public RNA-seq data. The genes are located on the right, and the tissues are indicated at the bottom of each column. The colour bar represents the expression values. S0-S38: developing seed from 0 to 38 DAP (day after pollination); Em10-Em38: developing embryo from 10 to 38 DAP; En6-En38: developing endosperm from 6 to 38 DAP

**Fig. 4:**
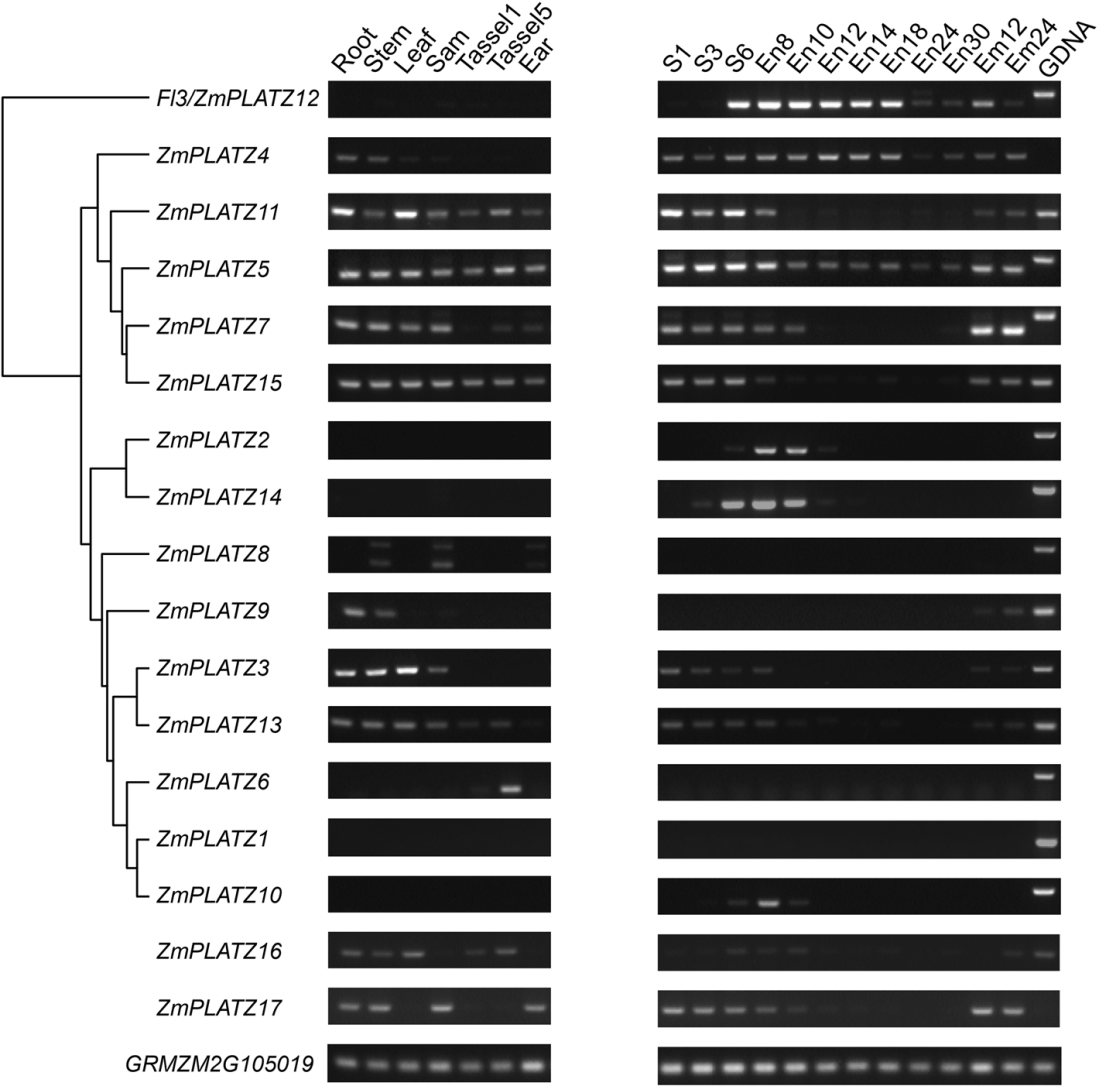
Expression patterns of *ZmPLATZ* genes by RT-PCR. The gene names are placed on the left, and the examined tissues are indicated on the top of each column. The phylogenetic tree was based on the RNA-seq data (B73 genome version 3). Since *ZmPLATZ16* and *ZmPLATZ17* were not annotated in B73 genome version 3, they were not included in the tree. Each *ZmPLATZ* gene was amplified with a specific primer pair for 32 cycles. The genomic DNA bands of *ZmPLATZ4* and *17* were not shown, due to their sizes being much larger than those of the cDNA bands. The GRMZM105019 gene was used as control. S1-S6: developing seed from 1 to 6 DAP; En8-En30: developing endosperm from 8 to 30 DAP; Em12-Em24: developing embryo from 12 to 24 DAP

The temporal and spatial expression patterns of the *PLATZ* genes in maize were investigated by analysing the transcripts using the public RNA-seq data [21] (Fig. 3) and RT-PCR (Fig. 4).

Three *PLATZs*, namely *11, 7* and *15*, were exhibited high and ubiquitous expression in all tissues except the developing endosperm. *PLATZ5* was expressed at varying levels in all tested tissues as shown by RT-PCR. but not in the public RNA-seq data. *PLATZ3* and *PLATZ13* exhibited similar expression patterns in root, stem, leaf, SAM and early seed, but *PLATZ3* had a higher expression level. The *PLATZ6* gene was specifically expressed in tassel, indicating that the function of this gene is involved in tassel development, The *PLATZ9* transcripts were only detected in root and stem. Transcript levels of *PLATZ4* were much higher in the developing endosperm than those in other tissues. However, *PLATZ4* was more ubiquitously expressed than *Fl3 (PLATZ12)* which expression was only detected at a high level in endosperm and at a weak level in the embryo (Fig. 4). Two other *PLATZs*, *2* and *14*, were expressed between 8 and 10 DAP in the endosperm, coincident with initiation of the endosperm filling. *PLATZ10* was weakly but specifically expressed in endosperm at 8 DAP. These four PLATZs might all be involved in maize endosperm development and storage reserve synthesis. We failed to clone *ZmPLATZ1* and *ZmPLATZ8* cDNAs from any tissue, most likely because they are only expressed in a highly differentiated tissue that was not investigated in the current study or under a special condition.

According to their expression levels and patterns [21], maize *PLATZ* genes could be clustered into two categories and *Fl3 (PLATZ12)* appeared as an out-group branch for its highest and specific expression in endosperm. The first category was composed of five genes (*PLATZ4*, *PLATZ5*, *PLATZ11, PLATZ7* and *PLATZ15*) with high and more ubiquitous expression levels, suggesting comprehensive roles in plant growth and development. The second category included other *PLATZs* of which the expression levels were relatively low and specific. ZmPLATZ16 and ZmPLATZ17 have not been included in either of the two clusters due to being missing in the B73 genome version 3.

### Subcellular localization of ZmPLATZs

**Fig. 5:**
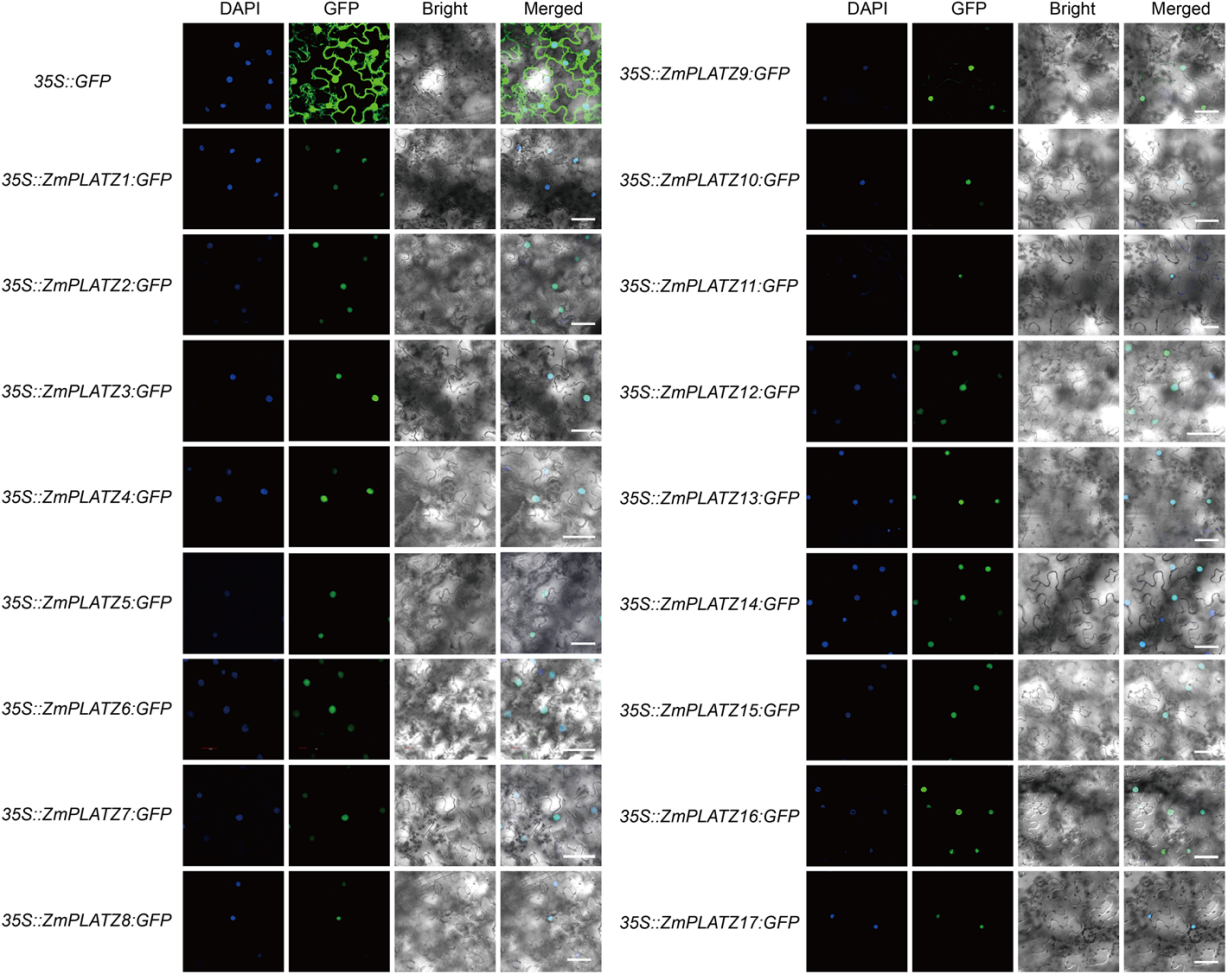
Subcellular localization of ZmPLATZs. The *GFP* gene was fused to the C-terminal of each ZmPLATZ. The constructs were transiently expressed in *N. benthamiana* leaves via *Agrobacteria* infiltration. Scale bars = 50 μm

The nuclear localization signal (NLS) could be predicted using wolf-psort (https://psort.hgc.jp/) or PredictNLS (https://rostlab.org/owiki/index.php/PredictNLS). A NLS was not identified in the FL3 (ZmPLATZ12) protein by online software, although the FL3-GFP fused protein is localized in nuclei [11]. To determine the subcellular localization of other members, each PLATZ protein was fused to green fluorescent protein (GFP). Because of the failure to amplify *ZmPLATZ1* and *ZmPLATZ8* cDNAs in any investigated tissue, their coding sequences were artificially synthesized (See methods). The free GFP was used as the control. The constitutive 35S promoter drove all gene cassettes. We transiently expressed the resulting constructs in tobacco leaves. All signals of the fused proteins including those of *35S::PLATZ1:GFP* and *35S::PLATZ8:GFP* were localized in nuclei, consistent with their predicted function as TFs, whereas the control *35S:GFP* was detected both in nuclei and the cytoplasm (Fig. 5).

### The protein-protein interaction of ZmPLATZs and RNAPIII

**Fig. 6:**
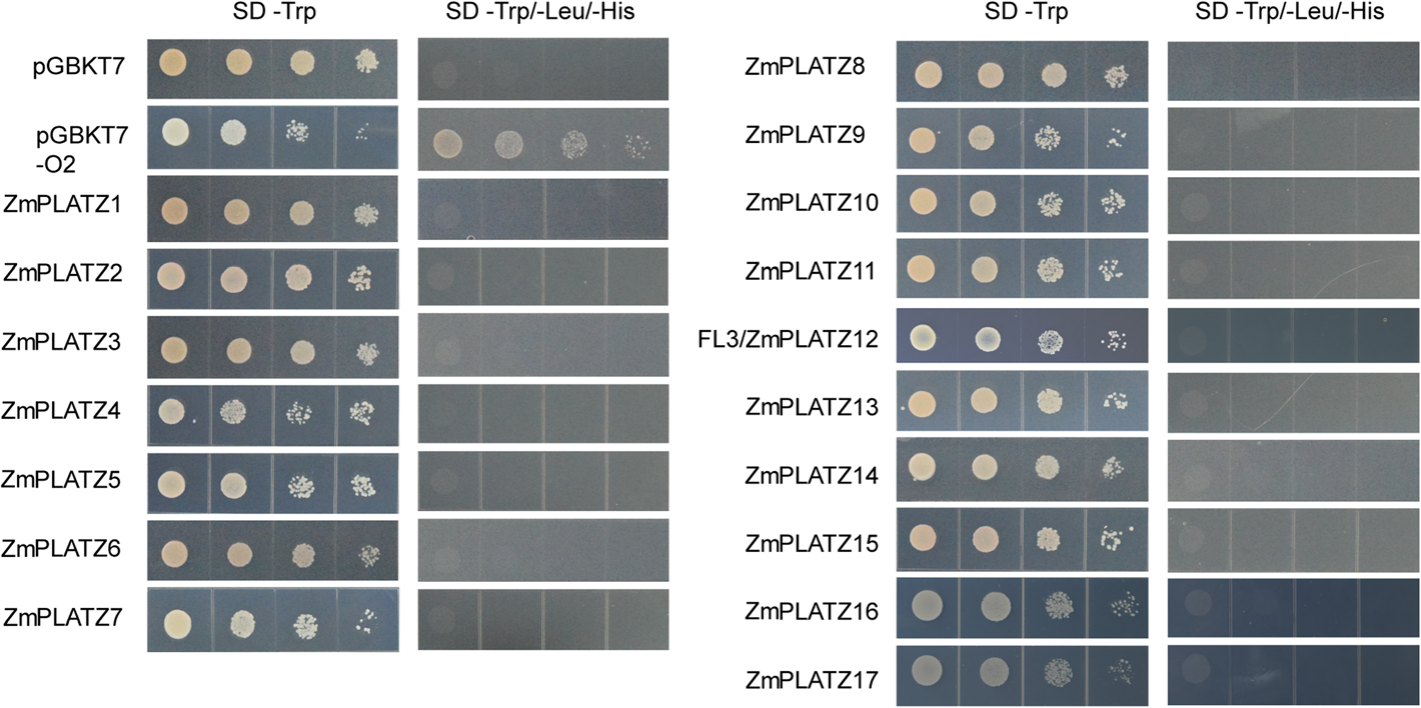
Auto-activation assay of ZmPLATZs in yeast Each ZmPLATZ and the endosperm-specific transcription factor O2 as the positive control were fused to the C-terminal of GAL4-BD. The resulting constructs pBD-PLATZs and pBD-O2 were transformed into Y2HGold and selected on the medium plates (SD/−Trp). Then, the transformed yeast colonies were grown on the selection medium plates (SD/−Trp/-His/−Ade)

**Fig. 7:**
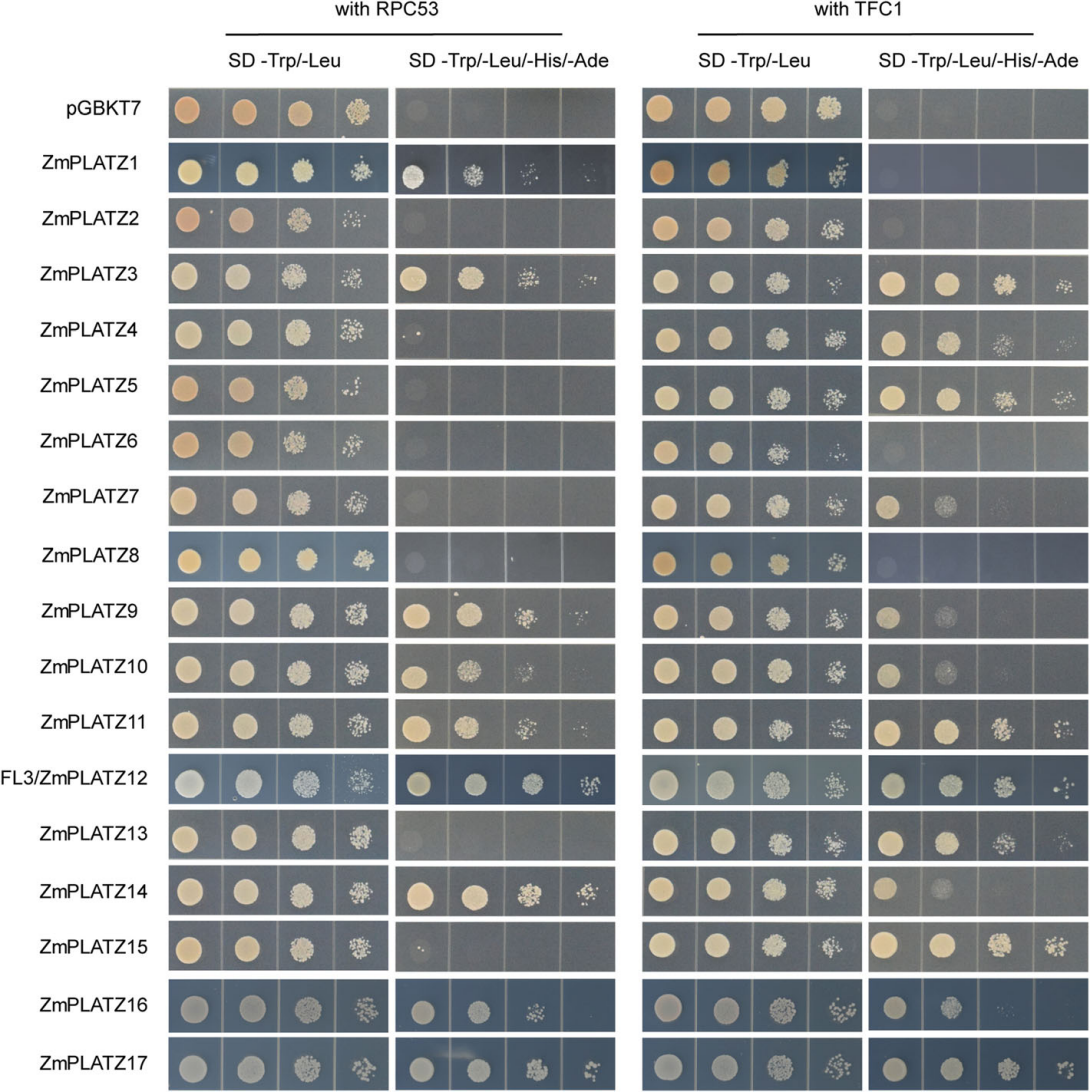
The protein-protein interaction assay of ZmPLATZs and RPC53/TFC1 by yeast two-hybrid assay. Constructs of pAD-RPC53/TFC1 and pBD-PLATZs were transformed into Y2HGold and selected on the medium plates (SD/−Trp/−Leu). Then, the transformed yeast colonies were grown on the selection medium plates (SD/−Trp/−Leu/-His/−Ade)

Previously, FL3 (ZmPLATZ12) was shown to have protein-protein interaction with RNAPIII subunits RPC53 and TFC1, but this protein was not found to have no intrinsic activation properties by using the yeast transactivation assay [11]. We then investigate other fused BD-ZmPLATZ proteins whether they were able bind to GAL4 upstream activating sequences (GALUAS) and activate transcription of the lacZ reporter gene. In contrast to the Opaque 2 (O2) control, an endosperm-specific bZIP TF for regulation of the storage-protein zein gene expression, none of PLATZs showed intrinsic activation properties (Fig. 6). Therefore, ZmPLATZs could be used to verify protein-protein interaction with yeast two-hybrid. We also tested whether other PLATZs could interact with RPC53 and TFC1. ZmPLATZ1 only interacted with RPC53, and ZmPLATZ4, ZmPLATZ5, ZmPLATZ7, ZmPLATZ13 and ZmPLATZ15 only interacted with TFC1. Similar to FL3 (ZmPLATZ12), ZmPLATZ3, ZmPLATZ9, ZmPLATZ10, ZmPLATZ11, ZmPLATZ14, ZmPLATZ16 and ZmPLATZ17 interacted with both. However, PLATZ2, PLATZ6 and PLATZ8 did not have a protein-protein interaction with RPC53 or TFC1 (Fig. 7). Collectively, these results indicate that PLATZ proteins are generally involved in modulation of RNAPIII-mediated transcription in different tissues.

### Phylogenetic analysis of PLATZ proteins in maize, rice and Arabidopsis

**Fig. 8:**
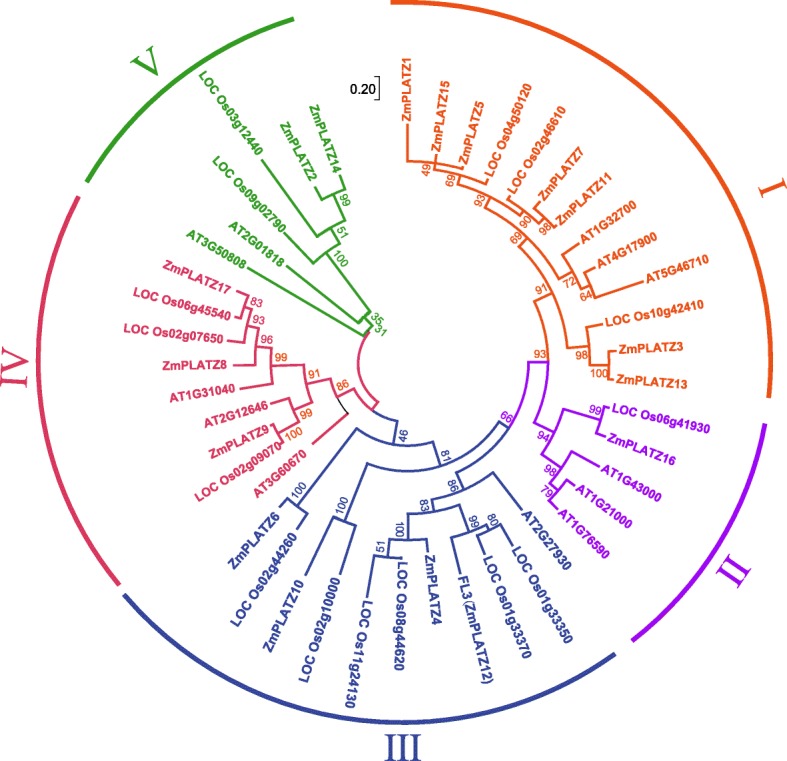
Phylogenetic analysis of ZmPLATZs, OsPLATZs and AtPLATZs. Maximum likelihood phylogenetic tree summarizes the evolutionary relationships among PLATZs. The numbers under the branches refer to the bootstrap values of the maximum likelihood phylogenetic tree. The length of the branches is proportional to the amino acid variation rates

We identified 17 ZmPLATZs from the maize genome. To explore the evolutionary conservation of PLATZ proteins in other species, we used the FL3 (ZmPLATZ12) protein sequence to blast against the rice (japonica cultivar-group, taxid: 39947, E-value ≤8e-18) and *Arabidopsis thaliana* (taxid: 3702, E-value ≤2e-05) reference protein databases. A total of 15 and 12 unique protein sequences were identified in rice and *Arabidopsis* databases, respectively (Additional file 7: File S3). To investigate the phylogenetic relationships among PLATZ proteins, we constructed a phylogenetic tree of the 17 ZmPLATZs, 15 OsPLATZs and 12 AtPLATZs. The maximum likelihood method was used to construct the phylogenetic tree using Clustal W and MEGA 7.0 (Fig. 8 and Additional file 8: Figure S4).

We divided the 44 PLATZ proteins into 5 subfamilies, designated I, II, III, IV and V based on the primary amino acid sequence. We noted that each subfamily included maize, rice and *Arabidopsis* members. Subfamily I was corresponding to clade1 of the phylogenetic tree of ZmPLATZs and contained a conserve domain (MAID-x_4–8_-L-x_4_-R-x_4–5_-GGG) in N-terminal (Additional file 8: Figure S4). Some ZmPLATZ members had OsPLATZ homologues with high bootstrap support (> 90%), such as ZmPLATZ9 and LOC Os02g09070, ZmPLATZ16 and LOC Os06g41930, and ZmPLATZ6 and LOC Os02g44260, indicating that these members are evolutionarily conserved in the grass family. Some ZmPLATZ members had two OsPLATZ homologues, such as LOC Os01g33350 and LOC Os01g33370 with ZmPLATZ12 and LOC Os08g44620 and LOC Os11g24130 with ZmPLATZ4. The close genome locations and similar expression patterns of LOC Os01g33350 and LOC Os01g33370 (http://rice.plantbiology.msu.edu/cgi-bin/ORF_infopage.cgi) indicated the two *OsPLATZ* genes resulted from gene duplication after the split with speciation of maize and rice*.*

## Discussion

### PLATZ proteins belong to a novel TF family interacting with RNAPIII

In a genome-wide screen of PLATZ proteins in the maize B73 genome version 3 and 4, we identified 17 complete members that all harboured the conserved PLATZ domain. Among the members, the expression of 15 *ZmPLATZs* was confirmed in variant tissues. The coding sequences of *ZmPLATZ1* and *ZmPLATZ8* were artificially synthesized for the following research. All ZmPLATZ proteins located to nuclei. Based on the random binding site selection (RBSS) experiment, A/T-rich sequences were recognized by FL3 (ZmPLATZ12). All members, except for ZmPLATZ2, ZmPLATZ6 and ZmPLATZ8, had a protein-protein interaction with either RPC53 or TFC1 or both (Fig. 7). This finding indicates that ZmPLATZ proteins are generally involved in modulation of RNAPIII transcription.

Although the gain-of-function mutant *fl3* shows severe defects in endosperm development and storage reserve filling, the knockout and knockdown mutations of this gene do not cause an apparent floury phenotype [11]. In addition to *FL3 (ZmPLATZ12)*, *ZmPLATZ2*, *ZmPLATZ4*, *ZmPLATZ10* and *ZmPLATZ14* were also expressed in the developing endosperm (Fig. 4). ZmPLATZ4 interacted with TFC1, and ZmPLATZ10/14 interacted with RPC53 and TFC1. One could envision that the three RNAPIII-interacting ZmPLATZs have redundant function with FL3 in the maize endosperm. Thus, creation of a series of double, triple and quadruple mutants of *ZmPLATZ4*, *ZmPLATZ10*, *Fl3* (*ZmPLATZ12)* and *ZmPLATZ14* will be an effective approach to overcome the functional redundancy.

### Classification and phylogenetic analysis of the plant-specific PLATZ family

A comparable number of *PLATZ* genes were identified in rice (15) and *Arabidopsis* (12), although the maize genome size (2300 Mb) [25] is ~ 5.3- and ~ 18.4-fold larger than that of rice (430 Mb) [26] and *Arabidopsis* (125 Mb) [27]. This huge discrepancy could be explained by a much higher percentage of transposons in the maize genome. The number of *PLATZ* genes is apparently conserved across different plant genomes.

The phylogenetic comparison of the PLATZ proteins was conducted in maize, rice and *Arabidopsis*, and their evolutionary relationships within and among the different species were investigated for the first time. Because of low identity between ZmPLATZs and AtPLATZs (the lowest at only 27%), the bootstrap values of some outer nodes were low; nevertheless, the internal nodes had more credible bootstrap values. The 44 PLATZ members from maize, rice and *Arabidopsis* were divided into 5 subfamilies (Fig. 8). The ML phylogenetic tree constructed by the 17 ZmPLATZ proteins could be divided into three branches (Fig. 2). Subfamily I of the total ML tree corresponding to clade1 of the phylogenetic tree of ZmPLATZs, contained a conserved domain (MAID-x_4–8_-L-x_4_-R-x_4–5_-GGG) in N-terminal. Meanwhile, the internal nodes between the two trees were comparable. For example, the branch of ZmPLATZ12 (FL3) and ZmPLATZ4 in the maize ML tree was also included in subfamily III in the total ML tree, and the branch of ZmPLATZ2 and ZmPLATZ14 in the maize ML tree was included in subfamily V in the total ML tree. Moreover, the subfamily III included LOC Os01g33350, LOC Os01g33370 and ZmPLATZ12, which display a similar expression pattern during rice and maize endosperm development, indicating a conserved function of the three orthologous PLATZ members in grass.

## Conclusions

In conclusion, we identified and cloned 17 *PLATZ* genes in the maize genome and found that seven PLATZs interacted with both RPC53 and TFC1. Our findings indicate that ZmPLATZ proteins are generally involved in the modulation of RNAPIII-mediated small non-coding RNA transcription.

## Additional files

Additional file 1: **Table S1.** Primer list. (XLSX 14 kb)

Additional file 2: **File S1.** cDNA sequences of 17 ZmPLATZs. (TXT 12 kb)

Additional file 3: **Figure S1.** ZmPLATZ4&9&11&14&17 cDNA sequence alignment. Sequence alignment of *ZmPLATZ4&9&11&14&17* CDS from cloned and predicted. (PDF 191 kb)

Additional file 4: **Figure S2.** The PLATZ6 cDNA sequence alignment. Sequence alignment of *ZmPLATZ6* CDS from cloned and predicted. (PDF 518 kb)

Additional file 5: **File S2.** Protein sequences of 17 ZmPLATZs. (TXT 4 kb)

Additional file 6: **Figure S3.** The amino acid sequence alignment of ZmPLATZs. Amino Acid Sequence Alignment of ZmPLATZs Black shaded amino acids represent identical amino acid residues and gray ones indicate the similar amino acid residues. (PDF 1843 kb)

Additional file 7: **File S3.** Protein sequences of AtPLATZs and OsPLATZs. (TXT 4 kb)

Additional file 8: **Figure S4.** The amino acid sequence alignment of AtPLATZs, OsPLATZs and ZmPLATZs. Amino Acid Sequence Alignment of ZmPLATZs, AtPLATZs and OsPLATZs. Black shaded amino acids represent identical amino acid residues and gray ones indicate the similar amino acid residues. (PDF 5870 kb)

## Abbreviations

DAP: Day after pollination
NLS: Nuclear localization signal
PLATZ: Plant AT-rich sequence and zinc-binding protein
RBSS: the random binding site selection
RNAPIII: RNA polymerase III
RPC53: RNA polymerase III subunit 53
TFC1: Transcription factor class C 1
