# Supplementary material for: Genome-wide analysis of the plant-specific PLATZ proteins in maize and identification of their general role in interaction with RNA polymerase III complex
Source: BMC Plant Biol. 2018 Oct 5;18:221. doi: 10.1186/s12870-018-1443-x (PMC6173924; doi:10.1186/s12870-018-1443-x)
Supplement: Supplementary file 3 — Figure S1. ZmPLATZ4&9&11&14&17 cDNA sequence alignment. Sequence alignment of ZmPLATZ4&9&11&14&17 CDS from cloned and predicted. (PDF 191 kb) [file 12870_2018_1443_MOESM3_ESM.pdf]

```
                              10        20        30        40        50        60        70
                      ....|....|....|....|....|....|....|....|....|....|....|....|....|....|
ZmPLATZ4(cloned)      ATGATGCCGCGGCGCGTGGCGTCGGTGCCGGTGCCCGACTGGCTGGAGGCGCTGCTGGCCACGCGCTTCT
ZmPLATZ4(predicted)   ATGATGCCGCGGCGCGTGGCGTCGGTGCCGGTGCCCGACTGGCTGGAGGCGCTGCTGGCCACGCGCTTCT

                              80        90       100       110       120       130       140
                      ....|....|....|....|....|....|....|....|....|....|....|....|....|....|
ZmPLATZ4(cloned)      TCCTCGCCTGCGCCGCGCACCCGGCGTCCCCGCGCAACGAGTGCAACATGTTCTGCCTCGACTGCACGGG
ZmPLATZ4(predicted)   TCCTCGCCTGCGCCGCGCACCCGGCGTCCCCGCGCAACGAGTGCAACATGTTCTGCCTCGACTGCACGGG

                             150       160       170       180       190       200       210
                      ....|....|....|....|....|....|....|....|....|....|....|....|....|....|
ZmPLATZ4(cloned)      CGCGCCGCCGCCGCCGCCGCCGGCCTTCTGCTACTACTGCCGCGCGCACCGACACTCGTCGCACCGCGTC
ZmPLATZ4(predicted)   CGCGCCGCCGCCGCCGCCGCCGGCCTTCTGCTACTACTGCCGCGCGCACCGACACTCGTCGCACCGCGTC

                             220       230       240       250       260       270       280
                      ....|....|....|....|....|....|....|....|....|....|....|....|....|....|
ZmPLATZ4(cloned)      ATCCAGATAAGGCGGTCCTCCTACCACGACGTGGTCCGCGTGTCGGAGGTGGAGGACGTGCTCGACATCT
ZmPLATZ4(predicted)   ATCCAGATAAGGCGGTCCTCCTACCACGACGTCGTCCGCGTGTCGGAGGTGGAGGACGTGCTCGACATCT

                             290       300       310       320       330       340       350
                      ....|....|....|....|....|....|....|....|....|....|....|....|....|....|
ZmPLATZ4(cloned)      CCGGCGTGCAGACCTACGTTATCAACAGCGCCCGGGTGCTCTTCCTCAACGAGAGGCCCCAGCCGCGCGG
ZmPLATZ4(predicted)   CCGGCGTGCAGACCTACGTCATCAACAGCGCCCGGGTGCTCTTCCTCAACGAGAGGCCCCAGCCGCGCGG

                             360       370       380       390       400       410       420
                      ....|....|....|....|....|....|....|....|....|....|....|....|....|....|
ZmPLATZ4(cloned)      CGCCGGCGCGGCCGCGGGCAAGGCCGCCGCGTCCCCCTACAACTGCGAGATCTGCGGCCGCGCGCTGCTC
ZmPLATZ4(predicted)   CGCCGGCGCGGCCGCGGGCAAGGCCGCCGCGTCCCCCTACAACTGCGAGATCTGCGGCCGCGCGCTGCTC

                             430       440       450       460       470       480       490
                      ....|....|....|....|....|....|....|....|....|....|....|....|....|....|
ZmPLATZ4(cloned)      GACCCCTTCCGCTTCTGCTCGCTCGGATGCAAGCTGGTGGACACCAAGAGGAGCAACGGCCACGCGGCTA
ZmPLATZ4(predicted)   GACCCCTTCCGCTTCTGCTCGCTCGGATGCAAGCTGGTGGACACCAAGAGGAGCAACGGCCACGCGGCTA

                             500       510       520       530       540       550       560
                      ....|....|....|....|....|....|....|....|....|....|....|....|....|....|
ZmPLATZ4(cloned)      GTTCCGCGGACGGAGGCGGCGGCGGCGGCGGCGCAGCCAGTGGCAATGACGAGACGACGGAGGCCGGCGG
ZmPLATZ4(predicted)   GTTCCGCGGACGGAGGCGGCGGCGGCGGCGGCGCAGCCAGTGGCAATGACGAGACGACGGAGGCCGGCGG

                             570       580       590       600       610       620       630
                      ....|....|....|....|....|....|....|....|....|....|....|....|....|....|
ZmPLATZ4(cloned)      GAGCAAGAACGGCCCGGGGGCGCGGCCGCACGGACGGCGGCGGAAGGGGACCCCACACCGCGCGCCCTTC
ZmPLATZ4(predicted)   GAGCAAGAACGGCCCGGGGGCGCGGCCGCACGGACGGCGGCGGAAGGGGACCCCACACCGCGCGCCCTTC

                      ....|....
ZmPLATZ4(cloned)      TGGTCCTGA
ZmPLATZ4(predicted)   TGGTCCTGA
```

```
                           10        20        30        40        50        60        70
                  ....|....|....|....|....|....|....|....|....|....|....|....|....|....|
ZmPLATZ9(cloned)     ATGATCATGCAGGCAATGTGGAAGCCAGGATGGCTAGAGGCCCTTGACACACAGAAGTTCTTCGTAGCAT
ZmPLATZ9(predicted)  ATGATCAT---GGCAATGTGGAAGCCAGGATGGCTAGAGGCCCTTGACACACAGAAGTTCTTCGTAGCAT

                           80        90       100       110       120       130       140
                  ....|....|....|....|....|....|....|....|....|....|....|....|....|....|
ZmPLATZ9(cloned)     GCTCTTTCCATGAGCATGCCAAGAAGAACGAGAAGAACATCTGTTGCCTTGACTGCTGCACTAGCATCTG
ZmPLATZ9(predicted)  GCTCTTTCCATGAGCATGCCAAGAAGAACGAGAAGAACATCTGTTGCCTTGACTGCTGCACTAGCATCTG

                          150       160       170       180       190       200       210
                  ....|....|....|....|....|....|....|....|....|....|....|....|....|....|
ZmPLATZ9(cloned)     CCCACACTGTGTGGCAGCACACCGTGCACACAGGCTCCTGCAGGTGCGGCGATACGTCTACCATGACGTT
ZmPLATZ9(predicted)  CCCACACTGTGTGGCAGCACACCGTGCACACAGGCTCCTGCAGGTGCGGCGATACGTCTACCATGACGTT

                          220       230       240       250       260       270       280
                  ....|....|....|....|....|....|....|....|....|....|....|....|....|....|
ZmPLATZ9(cloned)     GTCCGGCTGGAGGACCTGGAGAAGCTTATTGATTGCTCTAGTGTTCAGTCTTATACTATTAACAGCTCTA
ZmPLATZ9(predicted)  GTCCGGCTGGAGGACCTGGAGAAGCTTATTGATTGCTCTAGTGTTCAGTCTTATACTATTAACAGCTCTA

                          290       300       310       320       330       340       350
                  ....|....|....|....|....|....|....|....|....|....|....|....|....|....|
ZmPLATZ9(cloned)     AGGTTGTTTTCCTGAAGAAGAGACCACAGAATAGGCAATTCAAGGGTTCAGGGAATATCTGCACCTCCTG
ZmPLATZ9(predicted)  AGGTTGTTTTCCTGAAGAAGAGACCACAGAATAGGCAATTCAAGGGTTCAGGGAATATCTGCACCTCCTG

                          360       370       380       390       400       410       420
                  ....|....|....|....|....|....|....|....|....|....|....|....|....|....|
ZmPLATZ9(cloned)     CGACAGGAGCCTTCAAGAACCGTATTTCCACTGCTCTCTGGATTGCAAGGTAGAGTATATACTACGACAG
ZmPLATZ9(predicted)  CGACAGGAGCCTTCAAGAACCGTATTTCCACTGCTCTCTGGATTGCAAGGTAGAGTATATACTACGACAG

                          430       440       450       460       470       480       490
                  ....|....|....|....|....|....|....|....|....|....|....|....|....|....|
ZmPLATZ9(cloned)     AAGAAAAAATTGTCAGCATATTTGCGCCCATGCAAGACCTTGCAGCTTGGCCCTGATTTCTTCATTCCTC
ZmPLATZ9(predicted)  AAGAAAAAATTGTCAGCATATTTGCGCCCATGCAAGACCTTGCAGCTTGGCCCTGATTTCTTCATTCCTC

                          500       510       520       530       540       550       560
                  ....|....|....|....|....|....|....|....|....|....|....|....|....|....|
ZmPLATZ9(cloned)     ATGATGCTGATGACGACACAACTCACTCAACCCTTGTTGATGTTGATGAGCCCATGGGATCATCGGACTC
ZmPLATZ9(predicted)  ATGATGCTGATGACGACACAACTCACTCAACCCTTGTTGATGTTGATGAGCCCATGGGATCATCGGACTC

                          570       580       590       600       610       620       630
                  ....|....|....|....|....|....|....|....|....|....|....|....|....|....|
ZmPLATZ9(cloned)     GGAGAATTTGAGTGTGCCGTGCACAAATTTTGTTCGGAAAAAAACGGAGTGGACCATATATTTGTGCACGG
ZmPLATZ9(predicted)  GGAGAATTTGAGTGTGCCGTGCACAAATTTTGTTCGGAAAAAAACGGAGTGGACCATATATTTGTGCACGG

                          640       650       660       670       680       690       700
                  ....|....|....|....|....|....|....|....|....|....|....|....|....|....|
ZmPLATZ9(cloned)     TCTGCAAACAGAGTGTCTGAAGAAGACATGGCCACAAATATGAGCAGAAGGAAAGGGGTTCCTCAGAGAT
ZmPLATZ9(predicted)  TCTGCAAACAGAGTGTCTGAAGAAGACATGGCCACAAATATGAGCAGAAGGAAAGGGGTTCCTCAGAGAT

                          710
                  ....|....|....
ZmPLATZ9(cloned)     CGCCTTTGTGCTAA
ZmPLATZ9(predicted)  CGCCTTTGTGCTAA
```

```
                                10        20        30        40        50        60        70
                        ....|....|....|....|....|....|....|....|....|....|....|....|....|....|
ZmPLATZ11(cloned)       ATGGCGATAGACCACGCGGCGCGTCTCGGACTGACGAGCAGAGGTGCCACGGGAGGCGGCGGGTGCGGAG
ZmPLATZ11(predicted)    ATGGCGATAGACCACGCGGCGCGTCTCGGACTGACGAGCAGAGGTGCCACGGGAGGCGGCGGGTGCGGAG

                                80        90       100       110       120       130       140
                        ....|....|....|....|....|....|....|....|....|....|....|....|....|....|
ZmPLATZ11(cloned)       ACGACG---ACGCCGAGAACCGGCGGTGGCCGCCGTGGCTGAAGCCGCTGCTGTGCACGAGCTTCTTCGT
ZmPLATZ11(predicted)    ACGACGTCGACGCCGAGAACCGGCGGTGGCCGCCGTGGCTGAAGCCGCTGCTGTGCACGAGCTTCTTCGT

                               150       160       170       180       190       200       210
                        ....|....|....|....|....|....|....|....|....|....|....|....|....|....|
ZmPLATZ11(cloned)       CCAATGCAGGATCCACGCCGACGCGCACAAGAGCGAGTGCAACATGTACTGCCTCGACTGCATGGACGGC
ZmPLATZ11(predicted)    CCAATGCAGGATCCACGCCGACGCGCACAAGAGCGAGTGCAACATGTACTGCCTCGACTGCATGGACGGC

                               220       230       240       250       260       270       280
                        ....|....|....|....|....|....|....|....|....|....|....|....|....|....|
ZmPLATZ11(cloned)       GCGCTCTGCTCCCTCTGCCTCGCGCGCCACCGCGACCACCACTCCATCCAGATACGGAGGTCGTCGTACC
ZmPLATZ11(predicted)    GCGCTCTGCTCCCTCTGCCTCGCGCGCCACCGCGACCACCACTCCATCCAGATACGGAGGTCGTCGTACC

                               290       300       310       320       330       340       350
                        ....|....|....|....|....|....|....|....|....|....|....|....|....|....|
ZmPLATZ11(cloned)       ACGACGTGATCCGGGTGTCGGAGATACACAAGGTGCTGGACATCGCCGGCGTGCAGACGTACATCATCAA
ZmPLATZ11(predicted)    ACGACGTGATCCGGGTGTCGGAGATACACAAGGTGCTGGACATCGCCGGCGTGCAGACGTACATCATCAA

                               360       370       380       390       400       410       420
                        ....|....|....|....|....|....|....|....|....|....|....|....|....|....|
ZmPLATZ11(cloned)       CAGCGCGCGCGTGGTGTTCCTCAACGAGCGCCCGCAGCCTCGCCCGGGCAAGGGCGTCACCAACACCTGC
ZmPLATZ11(predicted)    CAGCGCGCGCGTGGTGTTCCTCAACGAGCGCCCGCAGCCTCGCCCGGGCAAGGGCGTCACCAACACCTGC

                               430       440       450       460       470       480       490
                        ....|....|....|....|....|....|....|....|....|....|....|....|....|....|
ZmPLATZ11(cloned)       GAGGTCTGCGAGCGCAGCCTCCTCGACTGCTTCCGCTTCTGCTCCCTCGGGTGCAAGATAGTGGGCACGG
ZmPLATZ11(predicted)    GAGGTCTGCGAGCGCAGCCTCCTCGACTGCTTCCGCTTCTGCTCCCTCGGGTGCAAGATAGTGGGCACGG

                               500       510       520       530       540       550       560
                        ....|....|....|....|....|....|....|....|....|....|....|....|....|....|
ZmPLATZ11(cloned)       CCCGTGGTTACCGTCCCAAGAAGAAGCACGGCAGCGGAGGCGGCGGCGGTAACAAGAGGAAGAGAGCGGC
ZmPLATZ11(predicted)    CGCGTGGTTACCGTCCCAAGAAGAAGCACGGCGGCGGAGGCGGCGGCGGTAACAAGAGGAAGAGAGCGGC

                               570       580       590       600       610       620       630
                        ....|....|....|....|....|....|....|....|....|....|....|....|....|....|
ZmPLATZ11(cloned)       GCTTAAGGACGTGCGCTCTGACTCGGAGGAGTCGTGCACCAGCACCAGCGGCGCCAGCAGCGACAAGAGC
ZmPLATZ11(predicted)    GCTTAAGGACGTGCGCTCTGACTCGGAGGAGTCGTGCACCAGCACCAGCGGCGCCAGCAGCGACAAGAGC

                               640       650       660       670       680       690       700
                        ....|....|....|....|....|....|....|....|....|....|....|....|....|....|
ZmPLATZ11(cloned)       AGCGTCGTGCAGAGCTTCTCGCCGTCGACCCCGCCGCCGGCCTCCGCTAGCTACCGCCGCCCAGGGAACA
ZmPLATZ11(predicted)    AGCGTCGTGCAGAGCTTCTCGCCGTCGACCCCGCCGCCGGCCTCCGCTAGCTACCGCCGCCCAGGGAACA
```

```
                                710       720       730       740       750
                        ....|....|....|....|....|....|....|....|....|....|....|....
ZmPLATZ11(cloned)       AGCGCCGGAAGGGCGTCCCGCACCGGTCCCCCTTCGGTAGCCTCATCGTCGAGTTCTAG
ZmPLATZ11(predicted)    AGCGCCGGAAGGGCGTCCCGCACCGGTCGCCCTTCGGTAGCCTCATCGTCGAGTTCTAG
```

```
                             10        20        30        40        50        60        70
                    ....|....|....|....|....|....|....|....|....|....|....|....|....|....|
ZmPLATZ14(cloned)    ATGTCTTGCATAGGTGGTTCCTCCTCTTCCACGGCGAGCAACAAGAGAAAGGGAAAGGAGATTGTGGTGG
ZmPLATZ14(predicted) ATGTCTTGCATAGGTGGTTCCTCCTCTTCCACGGCGAGCAACAAGAGAAAGGGAAAGGAGATTGTGGTGG

                             80        90       100       110       120       130       140
                    ....|....|....|....|....|....|....|....|....|....|....|....|....|....|
ZmPLATZ14(cloned)    AGGTGCCAGCTGTGGTGGAGGAGGAG---AAGCAGCAACAGCAGCACAGAAAGGGAAAGGAGGTTGCGTT
ZmPLATZ14(predicted) AGGTGTCAGCTGTGGTGGAGGAGGAGGAGAAGCAGCAGCAGCAGCAGAGAAAGGGAAAGGAGGTTGCGTT

                            150       160       170       180       190       200       210
                    ....|....|....|....|....|....|....|....|....|....|....|....|....|....|
ZmPLATZ14(cloned)    GGAGGAGGTGCCACTGCCAGCAGTGGCGGAGTCTTACGACGATTCAGATTTAGATTCGGGTTCGGGATGG
ZmPLATZ14(predicted) GGAGGAGGTGCCACTGCCTGCAGTGGCGGAGTCTTACGACGATTCAGATTTAGATTCGGGTTCTGGTTGG

                            220       230       240       250       260       270       280
                    ....|....|....|....|....|....|....|....|....|....|....|....|....|....|
ZmPLATZ14(cloned)    GATTTCTACGAGGAGGAGGACTCGAAGAAGAAGGAGCAAGAGAGGAAGGAAAAGAAACCTGCATGGCTGG
ZmPLATZ14(predicted) GACTTCTACGAGGAGGAGTACTCGGAGAAGAAGGAGCAAGAGAGGAAGGAAAAGAAACCTGCATGGCTGG

                            290       300       310       320       330       340       350
                    ....|....|....|....|....|....|....|....|....|....|....|....|....|....|
ZmPLATZ14(cloned)    ATACGCTGCTGAGAACCAAGTTCTGGGACCCGTGCAAGGAGCACGGGTCCAAGAACAGGGCAGACCAGTG
ZmPLATZ14(predicted) ATACGCTGCTGAGAACCAAGTTCTGGGACCCGTGCAAGGAGCACGGGTCCAAGAACAGGGCAGACCAGTG

                            360       370       380       390       400       410       420
                    ....|....|....|....|....|....|....|....|....|....|....|....|....|....|
ZmPLATZ14(cloned)    CATGTTCTGCCTCAGGTGCTCCAAGCTGAGCTGCCCTCGCTGTGTCCACGACCAGCCCGGCCACCGCCTC
ZmPLATZ14(predicted) CATGTTCTGCCTCAGGTGCTCCAAGCTGAGCTGCCCTCGCTGTGTCCACGACCAGCCCGGCCACCGCCTC

                            430       440       450       460       470       480       490
                    ....|....|....|....|....|....|....|....|....|....|....|....|....|....|
ZmPLATZ14(cloned)    CTCAAGATCCGCCGCTACGTCTACCGCTCCGTCGTCCACGCTTCCGACATGCAGGAGCTCGGCATCGACG
ZmPLATZ14(predicted) CTCAAGATCCGCCGCTACGTCTACCGCTCCGTCGTCCACGCTTCCGACATGCAGGAGCTCGGCATCGACG

                            500       510       520       530       540       550       560
                    ....|....|....|....|....|....|....|....|....|....|....|....|....|....|
ZmPLATZ14(cloned)    TCTCCAGGATACAGACATATGTCATCAATGCGAGAAAAGTGCTGCATCTGAGGCCCATGAACAGATCCAA
ZmPLATZ14(predicted) TCTCCAGGATACAGACATATGTCATCAATGCGAGAAAGGTGCTGCATCTGAGGCCCATGAACAGATCCAA

                            570       580       590       600       610       620       630
                    ....|....|....|....|....|....|....|....|....|....|....|....|....|....|
ZmPLATZ14(cloned)    GCATTTCAGGCCTCAAGCAGGGACACCTCGCTGCATAACCTGCAGGACCTGGCTGCGTAGCGCGCCCAAC
ZmPLATZ14(predicted) GCATTTCAGGCCTCAAGCAGGGACACCTCGCTGCATAACCTGCAGGACCTGGCTGCGTAGCGCGCCCAAC

                            640       650       660       670       680       690       700
                    ....|....|....|....|....|....|....|....|....|....|....|....|....|....|
ZmPLATZ14(cloned)    TTATTCTGCTCCCTTACCTGTGAGGAGGATGTTGATGTGTCGCAAGACGACTTCTCAGGGCCCGAAGCTG
ZmPLATZ14(predicted) TTATTTTGCTCCCTTACCTGTGAGGAGGATGTTGATGTGTCGCAAGATGACTTCTCAGGGCCCGAAGCTG

                            710       720       730       740       750       760       770
```

```
                          ....|....|....|....|....|....|....|....|....|....|....|....|....|....|
ZmPLATZ14(cloned)         AGCTCCGCTACAGGAGTTTCCAAGTGCATATGGCTGAACCTGCTGAGGAGCTGTTGCCTGATGACCCTGA
ZmPLATZ14(predicted)      AGCTCCGCTACAGGAGTTTCCAAGTGCATATGGCTGAACCTGCTGAGGAGCTGTTGCCTGATGACCCTGA

                                   780       790       800       810       820       830       840
                          ....|....|....|....|....|....|....|....|....|....|....|....|....|....|
ZmPLATZ14(cloned)         AGTTGAGCATGAGATCATGCCTGCACAAGTTGAGCCGCCACCACCGGCGGCCGCCGCCGCCGCAAATCAG
ZmPLATZ14(predicted)      AGTTGAGCATGAGATCATGCCTGCACAAGTTGAGCCGCCACCACTGGCGGCAGCCGCCGCCGCAAATCAG

                                   850       860       870       880       890       900
                          ....|....|....|....|....|....|....|....|....|....|....|....|...
ZmPLATZ14(cloned)         AACGTGTCACTCCGCAGGCGGGCGCGCAAGCAGGCAGCGCCATTGAGGGCGCCGTTCTTCTGA
ZmPLATZ14(predicted)      AACGTGTCACTCCGCAGGCGGGCGCGCAAGCAGGCAGCGCCATTGAGGGCGCCGTTCTTCTGA
```

```
                                10        20        30        40        50        60        70
                       ....|....|....|....|....|....|....|....|....|....|....|....|....|....|
ZmPLATZ17(cloned)      ATGGGGATGAGGCCCGGGTGGGTGGGCGGGCTGGTGGAGGAGAGCTTCTTCGTGGGGTGCGCGGCGCACG
ZmPLATZ17(predicted)   ATGGGGATGAGGCCCGGGTGGGTGGGCGGGCTGGTGGAGGAGAGCTTCTTCGTGGGGTGCGCGGCGCACG

                                80        90       100       110       120       130       140
                       ....|....|....|....|....|....|....|....|....|....|....|....|....|....|
ZmPLATZ17(cloned)      AGGACCGGAAGAAGAACGAGAAGAACATCTTCTGCCTGGGCTGCTGCGCCAGCATCTGCCCGCACTGCGC
ZmPLATZ17(predicted)   AGGACCGGAAGAAGAACGAGAAGAACATCTTCTGCCTGGGCTGCTGCGCCAGCATCTGCCCGCACTGCGC

                               150       160       170       180       190       200       210
                       ....|....|....|....|....|....|....|....|....|....|....|....|....|....|
ZmPLATZ17(cloned)      CCCCGCGCACCGCCACCACCTCCTGCTCCAGGTGCGGAGGTACGTGTACAATGACGTGGTGCGCCTTGAC
ZmPLATZ17(predicted)   CCCCGCGCACCGCCACCACCTCCTCCTCCAGGTGCGGAGGTACGTGTACAATGACGTGGTGCGCCTTGAC

                               220       230       240       250       260       270       280
                       ....|....|....|....|....|....|....|....|....|....|....|....|....|....|
ZmPLATZ17(cloned)      GACCTCGAGAGGCTCATCGACTGCTCCTTCGTCCAGCCCTACACGATCAACAGTGCCAAGGTGATCTTCC
ZmPLATZ17(predicted)   GACCTCGAGAGGCTCATCGACTGCTCCTTC---CAGCCCTACACGATCAACAGTGCCAAGGTGATCTTCC

                               290       300       310       320       330       340       350
                       ....|....|....|....|....|....|....|....|....|....|....|....|....|....|
ZmPLATZ17(cloned)      TCAAGCCGCGGCCGCAGTCCAGGCCCTTCAAGGGCTCTGGCAACGTCTGCTTGGCCTGTGACAGGATCCT
ZmPLATZ17(predicted)   TCAAGCCGCGGCCGCAGTCCAGGCCCTTCAAGGGCTCTGGCAACGTCTGCTTGGCCTGTGACAGGATCCT

                               360       370       380       390       400       410       420
                       ....|....|....|....|....|....|....|....|....|....|....|....|....|....|
ZmPLATZ17(cloned)      CCAGGAGCCCTTCCACTTCTGCTGCCTCTCCTGCAAGGTGGACCATGTGATGATGCAGGGCGGGGACCTG
ZmPLATZ17(predicted)   CCAGGAGCCCTTCCACTTCTGCTGCCTCTCCTGCAAGGTGGACCATGTGATGATGCAGGGCGGGGACCTG

                               430       440       450       460       470       480       490
                       ....|....|....|....|....|....|....|....|....|....|....|....|....|....|
ZmPLATZ17(cloned)      TCCAACATCCTCCTGTACGTGCCGGGCGGGCCGCCGGACCTGGGCTGCGGCTTCCCGCGCTTCGAGAACC
ZmPLATZ17(predicted)   TCCAACATCCTCCTGTACGTGCCGGGCGGGCCGCCGGACCTGGGCTGCGGCTTCCCGCGCTTCGAGAACC

                               500       510       520       530       540       550       560
                       ....|....|....|....|....|....|....|....|....|....|....|....|....|....|
ZmPLATZ17(cloned)      TCCGCTTCGACGACGACCCCGGGCAGTACGGGCAGGCCACGCCCAGCTCCATCCTCGAAGACCCCGTCGA
ZmPLATZ17(predicted)   TCCGCTTCGACGACGACCCCGGGCAGTACGGGCAGGCCACGCCCAGCTCCATCCTCGAAGACCCCGTCGA

                               570       580       590       600       610       620       630
                       ....|....|....|....|....|....|....|....|....|....|....|....|....|....|
ZmPLATZ17(cloned)      GCACGGGGGCAGCGCCAGCGCCAGCGGAGGATCCAGCAACGGCGGCTCGGCCAGGAACCACGCGCGGCGC
ZmPLATZ17(predicted)   GCACGGGGGCAGCGCCAGCGCCAGCGGAGGATCCAGCAACGGCGGCTCGGCCAGGAACCACGCGCGGCGC

                               640       650       660       670       680       690       700
                       ....|....|....|....|....|....|....|....|....|....|....|....|....|....|
ZmPLATZ17(cloned)      GACGTCGACGTCCCGACGAGGAAGAAGAAGAGCGGCGGGGGCGGAGGAGGGTTCTTCCCTCAGATCGTCC
ZmPLATZ17(predicted)   GACGTCGACGTCCCGACGAGGAAGAAGAAGAGCGGCGGGGGCGGAGGAGGGTTCTTCCCTCAGATCGTCC
```

```
                                   710       720       730       740       750
                         ....|....|....|....|....|....|....|....|....|....|...
ZmPLATZ17(cloned)        TCTCGCTCGGCAACAGGCGGAAGGGCGCGCCCCATAGGGCGCCGCTCGCGTAA
ZmPLATZ17(predicted)     TCTCGCTCGGCAACAGGCGGAAGGGCGCGCCCCATAGGGCGCCGCTCGCGTAA
```

Supplemental Fig1 Sequence alignment of ZmPLATZ4&9&11&14&17 CDS from cloned and predicted.
