# Supplementary material for: Genome-wide analysis of the plant-specific PLATZ proteins in maize and identification of their general role in interaction with RNA polymerase III complex
Source: BMC Plant Biol. 2018 Oct 5;18:221. doi: 10.1186/s12870-018-1443-x (PMC6173924; doi:10.1186/s12870-018-1443-x)
Supplement: Supplementary file 4 — Figure S2. The PLATZ6 cDNA sequence alignment. Sequence alignment of ZmPLATZ6 CDS from cloned and predicted. (PDF 518 kb) [file 12870_2018_1443_MOESM4_ESM.pdf]

```
                                 10        20        30        40        50        60        70        80
                        ....|....|....|....|....|....|....|....|....|....|....|....|....|....|....|....|
PLATZ6 GRMZM2G342691    ATGCATCCGGTGGCTGCGCGTGGCGCCCCGCACTGGCTGCGCGGCCTGCTATCGGAGGAGTTCTTCGACGCATGCGCCGC
PLATZ6 Zm00001d051376   ATGCATCCGGTGGCTGCGCGTGGCGCCCCGCACTGGCTGCGCGGCCTGCTATCGGAGGAGTTCTTCGACGCATGCGCCGC
PLATZ6 cloned           ATGCAGCCGGTGGCTGCGCGTGGCGCCCCGCATTGGCTGCGCGGCTTGCTATCGGAGGAGTTCTTCGACGCATGCGCAGC

                                 90       100       110       120       130       140       150       160
                        ....|....|....|....|....|....|....|....|....|....|....|....|....|....|....|....|
PLATZ6 GRMZM2G342691    GCACCCGGGGGAGCGCAAGAACGACAAGAACCACTTCTGCGTCGACTGCGCCGCGCCGCTGTGCCGCCACTGCCTCCCGC
PLATZ6 Zm00001d051376   GCACCCGGGGGAGCGCAAGAACGACAAGAACCACTTCTGCGTCGACTGCGCCGCGCCGCTGTGCCGCCACTGCCTCCCGC
PLATZ6 cloned           GCACCCGGGGGAGCGCAAGAACGACAAGAACCACTTCTGCGTCGACTGCGCCGCGCCGCTGTGCCGCCACTGCCTCCCGC

                                170       180       190       200       210       220       230       240
                        ....|....|....|....|....|....|....|....|....|....|....|....|....|....|....|....|
PLATZ6 GRMZM2G342691    ACGAACACGTCCACGACGTCCTCCAGATCTGGAAGTACGCGTCCTGCTTCGTCGTGCGCATCGACGACCTGAAGCTGTTC
PLATZ6 Zm00001d051376   ACGAACACGTCCACGACGTCCTCCAGATCTGGAAGTACGCGTCCTGCTTCGTCGTGCGCATCGACGACCTGAAGCTGTTC
PLATZ6 cloned           ACGAACACGTCCACGACGTCCTCCAGATCTGGAAGTACGCGTCCTGCTTCGTCGTGCGCATCGACGACCTGAAGCTGTTC

                                250       260       270       280       290       300       310       320
                        ....|....|....|....|....|....|....|....|....|....|....|....|....|....|....|....|
PLATZ6 GRMZM2G342691    GACTGCACCGGCATCCAGTCGCACACGGTGAGCGACCATGAGGTGGTGTTCCTGAACGAGCGTACGGCGAGGAAGCGGTC
PLATZ6 Zm00001d051376   GACTGCACCGGCATCCAGTCGCACACGGTGAGCGACCATGAGGTGGTGTTCCTGAACGAGCGTACGGCGAGGAAGCGGTC
PLATZ6 cloned           GACTGCACCGGCATCCAGTCGCACACGGTGAGCGACCATGAGGTGGTGTTCCTGAACGAGCGTACGGCGAGGAAGCGGTC

                                330       340       350       360       370       380       390       400
                        ....|....|....|....|....|....|....|....|....|....|....|....|....|....|....|....|
PLATZ6 GRMZM2G342691    CACGAGCGCCGAGAACCCCTGCGCCGCGTGCGCGCGACCGCTCCTCCCCGGCCACGACTACTGTTCACTCTTCTGCAAGC
PLATZ6 Zm00001d051376   CACGAGCGCCGAGAACCCCTGCGCCGCGTGCGCGCGACCGCTCCTCCCCGGCCACGACTACTGTTCACTCTTCTGCAAGG
PLATZ6 cloned           CACGAGTGCCGAGAACCCCTGCGCCGCGTGCGCGCGACCGCTACTCCCCGGCCACGACTACTGTTCACTCTTCTGCAAGG

                                410       420       430       440       450       460       470       480
                        ....|....|....|....|....|....|....|....|....|....|....|....|....|....|....|....|
PLATZ6 GRMZM2G342691    CGCGCCGCCGCAGCCCAAAACCATATCTCCCTTCCCGTAATTCCAGCCGTCGCGTGCGCACTAAATTCGAGTTGAAGCTC
PLATZ6 Zm00001d051376   TGAAGCATC-TGGGGGAGAGCGAG----CACGAGCTAAGGCGCGCGCTA-CGCGTGAGCCGGCAG---GAGGTG--GCTC
PLATZ6 cloned           TGAAGCATC-TGGGGGAGAGCGAG----CACGAGCTAAGGCGCGCGCTA-CGCGTGAGCCGGAAG---GAGGTG--GCTC

                                490       500       510       520       530       540       550       560
                        ....|....|....|....|....|....|....|....|....|....|....|....|....|....|....|....|
PLATZ6 GRMZM2G342691    CACCGCCTCATACTCAACGGCGGCTTGGCCGGTCCCGGCGCCTCCGCCGAAGACGTCGCCGACGCCCTCCGCGTCCCCTA
PLATZ6 Zm00001d051376   CCACGCCGGAGCCACA--GACCGGAGGAAGAGATCGTCGTCGTCGTCAGACGCGGGGCCGAG--CTGCAGCGGATCGTT
PLATZ6 cloned           CCACGCCGGAGCCACA--GACCGGAGGAAGAGATCGTCCTCGTCGTCAGACGCGGGGCCGAG--CTGCGGCGGATCGTT

                                570       580       590       600       610       620       630       640
                        ....|....|....|....|....|....|....|....|....|....|....|....|....|....|....|....|
PLATZ6 GRMZM2G342691    CCTGGAGTTCCGCCGCCAGAAGCGTGGCCCCTTCGTCGCCTCCGTCCGCCGCGCTCTCACCTCCATCCCCATCCCCTCCC
PLATZ6 Zm00001d051376   CCGGAAGCGAAGCCGGAAGCAGGCCGAGCCGGCACAGGCACCATTCCATTGA----------------------------
PLATZ6 cloned           CCGGAAGCGAAGCCGGAAGCAGGCCGAGCCGGCACAGGCACCATTCCATTGA----------------------------

                                650       660       670       680       690       700       710       720
                        ....|....|....|....|....|....|....|....|....|....|....|....|....|....|....|....|
PLATZ6 GRMZM2G342691    CATCCTCCTCTGACTCCTCCGACGATGGCTCCTCCGGCTCCCGCCGCCGCGGCCACCACGACGCCCACGCCACCACCGCC
PLATZ6 Zm00001d051376   --------------------------------------------------------------------------------
PLATZ6 cloned           --------------------------------------------------------------------------------

                                730       740       750       760       770       780       790       800
                        ....|....|....|....|....|....|....|....|....|....|....|....|....|....|....|....|
PLATZ6 GRMZM2G342691    TCCTCCTCCACCTCCGTATCCGACGCCGTTGCCCACCCGTCGCCACCAGCCCCTGCCTACGACGTCACCAAGTCCATGCT
PLATZ6 Zm00001d051376   --------------------------------------------------------------------------------
PLATZ6 cloned           --------------------------------------------------------------------------------
```

```
                                   810       820       830       840       850       860       870       880
                        ....|....|....|....|....|....|....|....|....|....|....|....|....|....|....|....|
PLATZ6 GRMZM2G342691    CCGCTCCCAGTACGCCGCGCAGACGCCCAAGCGGGGCCAGCAGCTGGAGATCGAGGTCGCGGCGGAGAAACTGCGCCGCC
PLATZ6 Zm00001d051376   --------------------------------------------------------------------------------
PLATZ6 cloned           --------------------------------------------------------------------------------

                                   890       900       910       920       930       940       950       960
                        ....|....|....|....|....|....|....|....|....|....|....|....|....|....|....|....|
PLATZ6 GRMZM2G342691    TCATCACGGCCGACGGTGGCGGCGGAGGCGATGCCAAGCCGGAGGCCGCCCCGTCTTCTGAAGGGTTCGGCAGAGGGGAA
PLATZ6 Zm00001d051376   --------------------------------------------------------------------------------
PLATZ6 cloned           --------------------------------------------------------------------------------

                                   970       980       990      1000      1010      1020      1030      1040
                        ....|....|....|....|....|....|....|....|....|....|....|....|....|....|....|....|
PLATZ6 GRMZM2G342691    GTCATATCTAGGGTGTCTGGAGGGGTTGTTGACATGCATTTGAGAAAGGTTAGGCACCTTGATACTTCTAAAAAGAGTGA
PLATZ6 Zm00001d051376   --------------------------------------------------------------------------------
PLATZ6 cloned           --------------------------------------------------------------------------------

                                  1050      1060      1070      1080      1090      1100      1110      1120
                        ....|....|....|....|....|....|....|....|....|....|....|....|....|....|....|....|
PLATZ6 GRMZM2G342691    ATTTCACAAGTTATTGCAACTGAAAGATGAACTATCAATCCATGTCAACTCAAGCAAGAGATATGGAACACAACTCAAGT
PLATZ6 Zm00001d051376   --------------------------------------------------------------------------------
PLATZ6 cloned           --------------------------------------------------------------------------------

                        ..
PLATZ6 GRMZM2G342691    AA
PLATZ6 Zm00001d051376   --
PLATZ6 cloned           --
```

Supplemental Fig2 Sequence alignment of ZmPLATZ6 CDS from cloned and predicted.
