# Supplementary material for: Genome-wide analysis of the plant-specific PLATZ proteins in maize and identification of their general role in interaction with RNA polymerase III complex
Source: BMC Plant Biol. 2018 Oct 5;18:221. doi: 10.1186/s12870-018-1443-x (PMC6173924; doi:10.1186/s12870-018-1443-x)
Supplement: Supplementary file 6 — Figure S3. The amino acid sequence alignment of ZmPLATZs. Amino Acid Sequence Alignment of ZmPLATZs Black shaded amino acids represent identical amino acid residues and gray ones indicate the similar amino acid residues. (PDF 1843 kb) [file 12870_2018_1443_MOESM6_ESM.pdf]

```
                   10        20        30        40        50        60        70
          ....|....|....|....|....|....|....|....|....|....|....|....|....|....|
ZmPLATZ5  ----------------------------------------------------------------------
ZmPLATZ15 ----------------------------------------------------------------------
ZmPLATZ1  MTKRCTNLACGMLPSMVGIGGRVGRCRLQLLGAFHSRPSPPSYPLRPSLPQSSAVIAVSNSCNIVDLHQR
ZmPLATZ7  ----------------------------------------------------------------------
ZmPLATZ11 ----------------------------------------------------------------------
ZmPLATZ3  ----------------------------------------------------------------------
ZmPLATZ13 ----------------------------------------------------------------------
ZmPLATZ16 ----------------------------------------------------------------------
ZmPLATZ4  ----------------------------------------------------------------------
ZmPLATZ12 ----------------------------------------------------------------------
ZmPLATZ10 ----------------------------------------------------------------------
ZmPLATZ6  ----------------------------------------------------------------------
ZmPLATZ2  -----------MSCIG--SSCTATNK--GKETVVEVHGAVAEEKQQ--VRNGEETAAEDDSGGSDPDSDS
ZmPLATZ14 -----------MSCIGGSSSSTASNKRKGKEIVVEVPAVVEEEKQQQQHRKGKEVALEEVPLPAVAESYD
ZmPLATZ9  ----------------------------------------------------------------------
ZmPLATZ8  ----------------------------------------------------------------------
ZmPLATZ17 ----------------------------------------------------------------------

                   80        90       100       110       120       130       140
          ....|....|....|....|....|....|....|....|....|....|....|....|....|....|
ZmPLATZ5  ----------MAIDDESP---LRVNPRGG-AMGGGERD----------GAENQRWPPWLKPLLGTSFFGQ
ZmPLATZ15 ----------MAIDDESP---IRVNSRGGGAMGGGECD----------GAENQRWPPWLKPLLGTSFFGQ
ZmPLATZ1  GVYSDVETLGTAVEPARRFTIYLVGPHRMRQ-GGGERDD----------AENQRWPPWLKPLLGTSFFSQ
ZmPLATZ7  ----------MAIDHATP---LGLKRRGAMG-GGGECDD---------GSDTRRWPPWLRPLLSASFFVQ
ZmPLATZ11 ----------MAIDHAAR---LGLTSRGATG-GGGCGDD---------DAENRRWPPWLKPLLCTSFFVQ
ZmPLATZ3  ----------MAIDHESPFKELRLKNRRIMG-GGGPDPEPEEEEE-VATAAYAGQWPRWLQPLLSARFFAH
ZmPLATZ13 ----------MAIDHESPFKELRLKNRRIMG-GGGPDPEPEEEEEEGVTAAYAERWPRWLQPLLSARFFAQ
ZmPLATZ16 -------MSAAAVALEEPAVQMVMVSVASPRSPAVRSE-------------EDLGPAWLRPLLGTSFFVP
ZmPLATZ4  ---------------------------MMPRRVAS-----------------VPVPDWLEALLATRFFLA
ZmPLATZ12 ----------------------------MKR---------------------ETMPSWLELLLATQFFTT
ZmPLATZ10 ----------------------MVSLREMARSDAERAP-----------------PAWLRALLETRFFDA
ZmPLATZ6  ----------------------------------------------MQPVAARGAPHWLRGLLSEEFFDA
ZmPLATZ2  ESDLDSESDWDSYSEEEEERQQQRKKKEKPKNKKKKKK---------QRRRQQQQPAWLITLLRTRFWEP
ZmPLATZ14 DSDLDSGSGWDFYEEEDS------KKKEQERKEKK--------------------PAWLDTLLRTKFWDP
ZmPLATZ9  -----------------------------------------------MIMQAMWKPGWLEALDTQKFFVA
ZmPLATZ8  -----------------------------------------------------------------MFSAN
ZmPLATZ17 ---------------------------------------------------MGMRPGWVGGLVEESFFVG
```

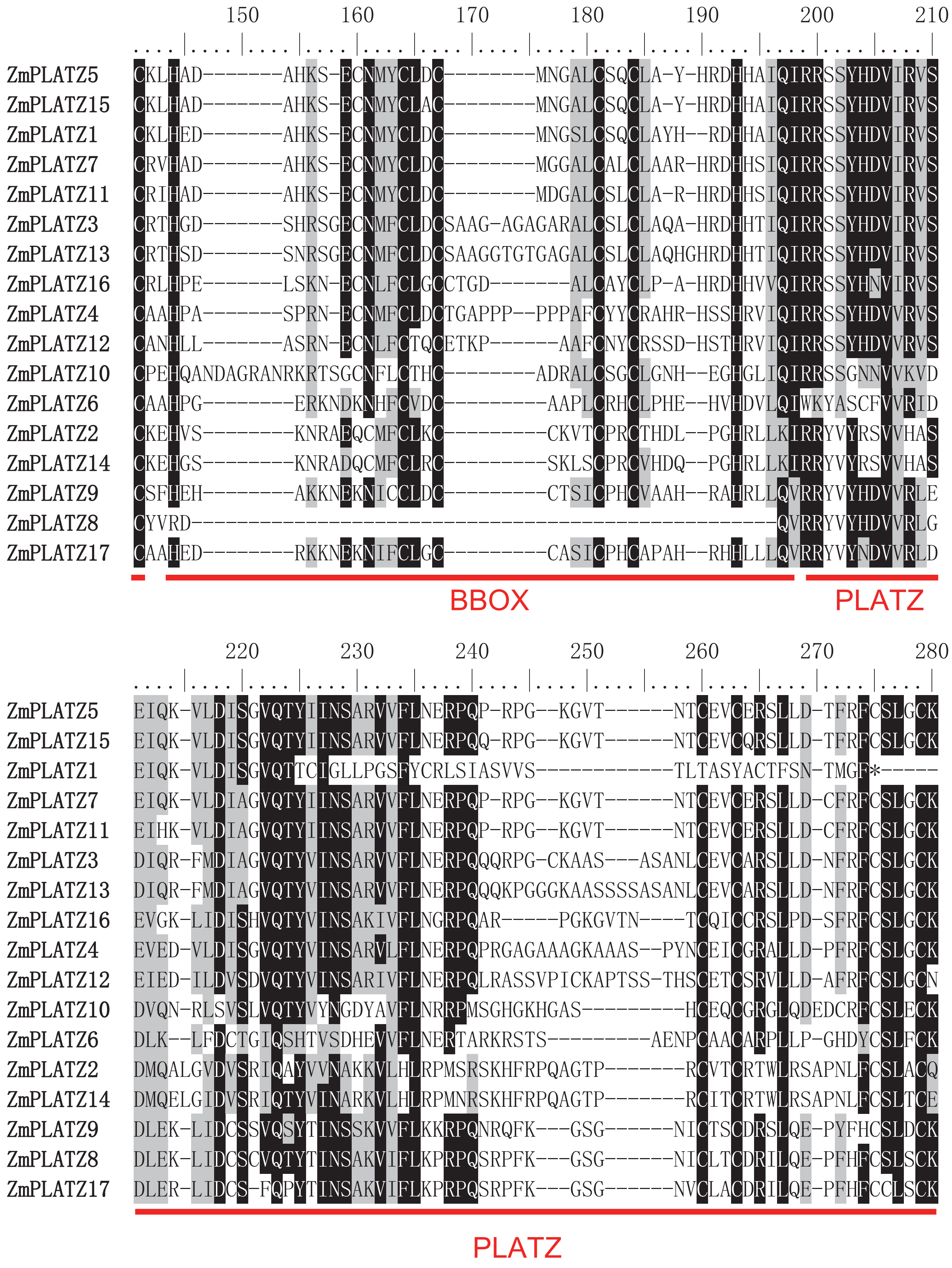
150 160 170 180 190 200 210
ZmPLATZ5 CKLHAD-------AHKS-ECNMYCLDC--------MNGALCSQCLA-Y-HRDHHAIQIRRSSYHDVIRVS
ZmPLATZ15 CKLHAD-------AHKS-ECNMYCLAC--------MNGALCSQCLA-Y-HRDHHAIQIRRSSYHDVIRVS
ZmPLATZ1 CKLHED-------AHKS-ECNMYCLDC--------MNGSLCSQCLAYH--RDHHAIQIRRSSYHDVIRVS
ZmPLATZ7 CRVHAD-------AHKS-ECNMYCLDC--------MGGALCALCLAAR-HRDHHSIQIRRSSYHDVIRVS
ZmPLATZ11 CRIHAD-------AHKS-ECNMYCLDC--------MDGALCSLCLA-R-HRDHHSIQIRRSSYHDVIRVS
ZmPLATZ3 CRTHGD-------SHRSGECNMFCLDCSAAG-AGAGARALCSLCLAQA-HRDHHTIQIRRSSYHDVIRVS
ZmPLATZ13 CRTHSD-------SNRSGECNMFCLDCSAAGGTGTGAGALCSLCLAQHGHRDHHTIQIRRSSYHDVIRVS
ZmPLATZ16 CRLHPE-------LSKN-ECNLFCLGCCTGD-------ALCAYCLP-A-HRDHHVVQIRRSSYHNVIRVS
ZmPLATZ4 CAAHPA-------SPRN-ECNMFCLDCTGAPPP--PPPAFCYYCRAHR-HSSHRVIQIRRSSYHDVVRVS
ZmPLATZ12 CANHLL-------ASRN-ECNLFCTQCETKP------AAFCNYCRSSD-HSTHRVIQIRRSSYHDVVRVS
ZmPLATZ10 CPEHQANDAGRANRKRTSGCNFLCTHC--------ADRALCSGCLGNH--EGHGLIQIRRSSGNNVVKVD
ZmPLATZ6 CAAHPG--------ERKNDKNHFCVDC---------AAPLCRHCLPHE--HVHDVLQIWKYASCFVVRID
ZmPLATZ2 CKEHVS--------KNRAEQCMFCLKC---------CKVTCPRCTHDL--PGHRLLKIRRYVYRSVVHAS
ZmPLATZ14 CKEHGS--------KNRADQCMFCLRC---------SKLSCPRCVHDQ--PGHRLLKIRRYVYRSVVHAS
ZmPLATZ9 CSFHEH--------AKKNEKNICCLDC---------CTSICPHCVAAH--RAHRLLQVRRYVYHDVVRLE
ZmPLATZ8 CYVRD---QVRRYVYHDVVRLG
ZmPLATZ17 CAAHED--------RKKNEKNIFCLGC---------CASICPHCAPAH--RHHLLLQVRRYVYNDVVRLD
BBOX
PLATZ
220 230 240 250 260 270 280
ZmPLATZ5 EIQK-VLDISGVQTYIINSARVVFLNERPQP-RPG--KGVT------NTCEVCERSLLD-TFRFCSLGCK
ZmPLATZ15 EIQK-VLDISGVQTYIINSARVVFLNERPQQ-RPG--KGVT------NTCEVCQRSLLD-TFRFCSLGCK
ZmPLATZ1 EIQK-VLDISGVQTTCIGLLPGSFYCRLSIASVVS------------TLTASYACTFSN-TMGF*-----
ZmPLATZ7 EIQK-VLDIAGVQTYIINSARVVFLNERPQP-RPG--KGVT------NTCEVCERSLLD-CFRFCSLGCK
ZmPLATZ11 EIHK-VLDIAGVQTYIINSARVVFLNERPQP-RPG--KGVT------NTCEVCERSLLD-CFRFCSLGCK
ZmPLATZ3 DIQR-FMDIAGVQTYVINSARVVFLNERPQQQRPG-CKAAS---ASANLCEVCARSLLD-NFRFCSLGCK
ZmPLATZ13 DIQR-FMDIAGVQTYVINSARVVFLNERPQQQKPGGGKAASSSSASANLCEVCARSLLD-NFRFCSLGCK
ZmPLATZ16 EVGK-LIDISHVQTYVINSAKIVFLNGRPQAR-----PGKGVTN----TCQICCRSLPD-SFRFCSLGCK
ZmPLATZ4 EVED-VLDISGVQTYVINSARVLFLNERPQPRGAGAAAGKAAAS--PYNCEICGRALLD-PFRFCSLGCK
ZmPLATZ12 EIED-ILDVSDVQTYVINSARIVFLNERPQLRASSVPICKAPTSS-THSCETCSRVLLD-AFRFCSLGCN
ZmPLATZ10 DVQN-RLSVSLVQTYVYNGDYAVFLNRRPMSGHGKHGAS---------HCEQCGRGLQDEDCRFCSLECK
ZmPLATZ6 DLK--LFDCTGIQSHTVSDHEVVFLNERTARKRSTS---------AENPCAACARPLLP-GHDYCSLFCK
ZmPLATZ2 DMQALGVDVSRIQAYVVNAKKVLHLRPMSRSKHFRPQAGTP-------RCVTCRTWLRSAPNLFCSLACQ
ZmPLATZ14 DMQELGIDVSRIQTYVINARKVLHLRPMNRSKHFRPQAGTP-------RCITCRTWLRSAPNLFCSLTCE
ZmPLATZ9 DLEK-LIDCSSVQSYTINSSKVVFLKKRPQNRQFK---GSG------NICTSCDRSLQE-PYFHCSLDCK
ZmPLATZ8 DLEK-LIDCSCVQTYTINSAKVIFLKPRPQSRPFK---GSG------NICLTCDRILQE-PFHFCSLSCK
ZmPLATZ17 DLER-LIDCS-FQPYTINSAKVIFLKPRPQSRPFK---GSG------NVCLACDRILQE-PFHFCCLSCK
PLATZ

```
                   290       300       310       320       330       340       350
          ....|....|....|....|....|....|....|....|....|....|....|....|....|....|
ZmPLATZ5  IVRTSGDFRI-RKKHAI--------------------------------------VAKKKREKKHAPQQK
ZmPLATZ15 IVGTSGDLRI-RKKQAV--------------------------------------VKKHQKKKKQ--QAQ
ZmPLATZ1  ----------------------------------------------------------------------
ZmPLATZ7  IVGTARGYRPGKKKHG---------------------------------------GGGGNKKRASPAPAL
ZmPLATZ11 IVGTARGYRP-KKKHGS--------------------------------------GGGGGNKRKR--AAL
ZmPLATZ3  VVGCS---------------------------------------------------PDAAKARSWLLRPA
ZmPLATZ13 VVGCS---------------------------------------------------PDAAKARNWLLRAA
ZmPLATZ16 LGGMQWDPSLTFAIRPKRG----------------------------------QGSGDDGGSGSDDSFSP
ZmPLATZ4  LVDTKRSNGHAASS------------------------------------------ADGGGGGG------
ZmPLATZ12 LRGLNMEAGMQAMV------------------------------------------GNNPRSNGMDHVAR
ZmPLATZ10 AKGIEDR-------------------------------------------------LDFSVSFAVDPNNF
ZmPLATZ6  VKHLG----------------------------------------------------ESEHELRRALRVS
ZmPLATZ2  GNVDVAQDDFSG--------------------------------------------PEAEVRYRSLQVQM
ZmPLATZ14 EDVDVSQDDFSG--------------------------------------------PEAELRYRSFQVHM
ZmPLATZ9  VEYILRQKKKLSAYLRPCK--------TLQLG----PDFFIPHDADDD---------TTHSTLVDVDEP-
ZmPLATZ8  VDHVMTQGGDLSNILQHYGAGGGGGGGTADPDRLAFPRFENLRVVDGSDLDDDVQVVTPDSTLEDPTNN-
ZmPLATZ17 VDHVMMQGGDLSNILLYVP------GGPPDLG-CGFPRFENLRFDDDP---GQYGQATPSSILEDPVEHG

                   360       370       380       390       400       410       420
          ....|....|....|....|....|....|....|....|....|....|....|....|....|....|
ZmPLATZ5  QHRGAADS----ADDDDDDSSTSTSGGS-DKSSVVQSFTPSTPPATTANSFRAG-KRRKGVPHRSPFGSL
ZmPLATZ15 QHRGAA------LDSEDDSSSTSTSRGS-DRSSVVQSFTPSTPPAT-ANSFRTG-KRRKGVPHRSPFGSL
ZmPLATZ1  ----------------------------------------------------------------------
ZmPLATZ7  KD----------VRSDSEDSCTSTSGASSDKSSVVQS---SPPPPTSASHRPPGNKRRKGIPHRSPLGSL
ZmPLATZ11 KD----------VRSDSEESCTSTSGASSDKSSVVQSFSPSTPPPASASYRRPGNKRRKGVPHRSPFGSL
ZmPLATZ3  VG------------GSGDGDSTSSSPLR--DAQKRQSFTPPTPQP---------AKRRKGIPHRAPFGSF
ZmPLATZ13 D---------------GDGSTTSSSAPR--NADRKLSFTPPTPQPT------LPTKRRKGIPHRAPFGSL
ZmPLATZ16 KKPRRMAGFDLGRFERPGGIRWSDDEGSRSNNGLITPGTPPINRCR--------PSRRKGIPHRAPFYG-
ZmPLATZ4  ------------------GAASGNDE-TTEAGGSKNG---PGARPH--------GRRRKGTPHRAPFWS*
ZmPLATZ12 IDNV--------------GSSTTNDQNSCNDKNYEEP---PPKRVA--------RHRRKGIPQRAPFF*-
ZmPLATZ10 SS-----------SGDDTESDDDEDSSYPSKFQKLETIPASSSKPVASGGQHSIGKKQY*----------
ZmPLATZ6  RK---------------EVAPTPEPQTGRKRSSSSSDAGPSCGGSFRKR------SRKQAEPAQAPFH*-
ZmPLATZ2  AEPS---------GAAADELPG-PEAAHEV-PAQVPPPPPP---AANQNASLRRRPRKQAAPERAPFF*-
ZmPLATZ14 AEP------------AEELLPDDPEVEHEIMPAQVEPPPPAAAAAANQNVSLRRRARKQAAPLRAPFF*-
ZmPLATZ9  ------MGSSDSENLSVPCTNFVRKKRSGPYICAR-SANRVSEEDMATN-----MSRRKGVPQRSPLC*-
ZmPLATZ8  ----AGGGSSDNG--TDDARRQVVVHGGGEAAKRKKGGGFLPQIVLSLGGGGGGGNRRKGAPHRSPLA*-
ZmPLATZ17 GSASASGGSSNGGSARNHARRDVDVPTRKKKSGGG-GGGFFPQIVLSLG------NRRKGAPHRAPLA--
```

	....|.
ZmPLATZ5	VVEF*-
ZmPLATZ15	MVEEF*
ZmPLATZ1	------
ZmPLATZ7	IVEL*-
ZmPLATZ11	IVEF*-
ZmPLATZ3	IVEY*-
ZmPLATZ13	IVEY*-
ZmPLATZ16	------
ZmPLATZ4	------
ZmPLATZ12	------
ZmPLATZ10	------
ZmPLATZ6	------
ZmPLATZ2	------
ZmPLATZ14	------
ZmPLATZ9	------
ZmPLATZ8	------
ZmPLATZ17	------

Supplemental Fig3 Amino Acid Sequence Alignment of ZmPLATZs.
Black shaded amino acids represent identical amino acid residues
and gray ones indicate the similar amino acid residues.
