# Supplementary material for: Genome-wide analysis of the plant-specific PLATZ proteins in maize and identification of their general role in interaction with RNA polymerase III complex
Source: BMC Plant Biol. 2018 Oct 5;18:221. doi: 10.1186/s12870-018-1443-x (PMC6173924; doi:10.1186/s12870-018-1443-x)
Supplement: Supplementary file 8 — Figure S4. The amino acid sequence alignment of AtPLATZs, OsPLATZs and ZmPLATZs. Amino Acid Sequence Alignment of ZmPLATZs, AtPLATZs and OsPLATZs. Black shaded amino acids represent identical amino acid residues and gray ones indicate the similar amino acid residues. (PDF 5870 kb) [file 12870_2018_1443_MOESM8_ESM.pdf]

```
                         10        20        30        40        50        60        70
                 ....|....|....|....|....|....|....|....|....|....|....|....|....|....|
ZmPLATZ1         MTKRCTNLACGMLPSMVGIGGRVGRCRLQLLGAFHSRPSPPSYPLRPSLPQSSAVIAVSNSCNIVDLHQR
ZmPLATZ5         ----------------------------------------------------------------------
ZmPLATZ15        ----------------------------------------------------------------------
LOC_Os04g50120   ----------------------------------------------------------------------
LOC_Os02g46610   ----------------------------------------------------------------------
ZmPLATZ7         ----------------------------------------------------------------------
ZmPLATZ11        ----------------------------------------------------------------------
AT1G32700        ----------------------------------------------------------------------
AT4G17900        ----------------------------------------------------------------------
AT5G46710        ----------------------------------------------------------------------
LOC_Os10g42410   ----------------------------------------------------------------------
ZmPLATZ3         ----------------------------------------------------------------------
ZmPLATZ13        ----------------------------------------------------------------------
LOC_Os06g41930   ----------------------------------------------------------------------
ZmPLATZ16        ----------------------------------------------------------------------
AT1G43000        ----------------------------------------------------------------------
AT1G21000        ----------------------------------------------------------------------
AT1G76590        ----------------------------------------------------------------------
AT2G27930        ----------------------------------------------------------------------
LOC_Os01g33350   ----------------------------------------------------------------------
LOC_Os01g33370   ----------------------------------------------------------------------
ZmPLATZ12        ----------------------------------------------------------------------
ZmPLATZ4         ----------------------------------------------------------------------
LOC_Os08g44620   ----------------------------------------------------------------------
LOC_Os11g24130   ----------------------------------------------------------------------
LOC_Os02g10000   ----------------------------------------------------------------------
ZmPLATZ10        ----------------------------------------------------------------------
LOC_Os02g44260   ----------------------------------------------------------------------
ZmPLATZ6         ----------------------------------------------------------------------
AT3G60670        ----------------------------------------------------------------------
LOC_Os02g09070   ----------------------------------------------------------------------
ZmPLATZ9         ----------------------------------------------------------------------
AT2G12646        ----------------------------------------------------------------------
AT1G31040        ----------------------------------------------------------------------
ZmPLATZ8         ----------------------------------------------------------------------
LOC_Os02g07650   ----------------------------------------------------------------------
LOC_Os06g45540   ----------------------------------------------------------------------
ZmPLATZ17        ----------------------------------------------------------------------
AT3G50808        ----------------------------------------------------------------------
AT2G01818        ----------------------------------------------------------------------
LOC_Os09g02790   ----------------------------------MLPSPDDSSSSSPSSKTNPSEKGEEIT--VVEEPIL
LOC_Os03g12440   --------------------------MLSKMHMKWFSFLEWPSATFASSFIKTQSSLAQYYLLMFVKIIE
ZmPLATZ2         -----------------------------------MSCIG--SSCTATNK--GKETVVEVHGAVAEEKQQ
ZmPLATZ14        -----------------------------------MSCIGGSSSSTASNKRKGKEIVVEVPAVVEEEKQQ
```

```
                        80        90        100       110       120       130       140
                    ....|....|....|....|....|....|....|....|....|....|....|....|....|....|
ZmPLATZ1            GVYSDVETLGTAVEPARRFTIYLVGPHRMRQGGGERDDAENQ---------------------------R
ZmPLATZ5            ---MAIDD-----ESP---LRVNPRGG-AMGGG-ERDGAENQ---------------------------R
ZmPLATZ15           ---MAIDD-----ESP---IRVNSRGGGAMGGG-ECDGAENQ---------------------------R
LOC_Os04g50120      ---MAIDD-----ESP---LRINTTRGGAMGGGGECDGAENQ---------------------------R
LOC_Os02g46610      ---MAIDH-----ASP---FSLK-NRGDMGGRGYEEEEVENQ---------------------------R
ZmPLATZ7            ---MAIDH-----ATP---LGLK-RRGAMGGGG[illegible]CDDGSDTR---------------------------R
ZmPLATZ11           ---MAIDH-----AAR---LGLT-SRGATGGGGGGDDDAENR---------------------------R
AT1G32700           -------------------------------MGA--EEETNK---------------------------T
AT4G17900           ---MAIED-----QEN---TIREIKPKNRRIMGAGGPEEEEN---------------------------R
AT5G46710           ---MAIEDY----ENPNREIKPKNRRFMEG---------ENQ----------------------------
LOC_Os10g42410      ---MAIDH-----ESPFKELRLKNRRI-MGGGG[illegible]EPE---EEE--------------------AVAHGEQ
ZmPLATZ3            ---MAIDH-----ESPFKELRLKNRRI-MGGGG[illegible]DPEPEEEEE-VA-----------------TAAYAGQ
ZmPLATZ13           ---MAIDH-----ESPFKELRLKNRRI-MGGGG[illegible]DPEPEEEEEEGV-----------------TAAYAER
LOC_Os06g41930      -----------MVMVGMMETSPDDGGGGEAMAA----AQQTEE-------------------------DM
ZmPLATZ16           -----------MSAAAVALEEPAVQMVMVSVASPRSPAVRSEE-------------------------DL
AT1G43000           ------------------MENDDV---------------------------------------------M
AT1G21000           -----------MGPMIRTEEEEDY---------------------------------------------T
AT1G76590           -----------MGPMMMRAEEDNYNL-------------------------------------------N
AT2G27930           --------------------------------------------------------------------ME
LOC_Os01g33350      ---------------------------------------MKG--------------------------EF
LOC_Os01g33370      ---------------------------------------MKGG-------------------------ES
ZmPLATZ12           ---------------------------------------MKR--------------------------ET
ZmPLATZ4            -----------------------------------MMPRRVAS-------------------------VP
LOC_Os08g44620      -----------------------------------MSEGAAGT-------------------------TR
LOC_Os11g24130      ----------------------------------------------------------------------
LOC_Os02g10000      -----------------------------------MAAGLAE--------------------------RE
ZmPLATZ10           -----------------------------MVSLREMARSDAE--------------------------RA
LOC_Os02g44260      ----------------------------------MQSPAVRG----------------------------
ZmPLATZ6            ----------------------------------MQPVAARG----------------------------
AT3G60670           -----------------------------------------------------------------MESGE
LOC_Os02g09070      ---------------------------------------------------------------MIMQAMW
ZmPLATZ9            ---------------------------------------------------------------MIMQAMW
AT2G12646           ------------------------------------------------------------------MGIQ
AT1G31040           -----------------------------------------------------MVREGEEEEEMMMMMAT
ZmPLATZ8            ----------------------------------------------------------------------
LOC_Os02g07650      -------------------------------------------------------------------MGM
LOC_Os06g45540      -------------------------------------------------------------------MGM
ZmPLATZ17           -------------------------------------------------------------------MGM
AT3G50808           ----------------------------------------------------------------------
AT2G01818           ---MNLSEKRRSEE--------------------------------------------------------
LOC_Os09g02790      --FDNNQEEDNSSNPLNLLADT---SEL-----FRNMDDDES----------------------------
LOC_Os03g12440      LCKLKGKETRAEPEVSSVSKASGGSSEHINKLPPPVPVQEEEE---------------------------
ZmPLATZ2            --VRNGEETAAEDDSGGSDPDSDSESDLDSESDWDSYSEEEEERQQQRKKKEKPNKKKKKKQRRRQQQQ
ZmPLATZ14           QQHRKGKEVALEEVPLPAVAESYDDSDLDSGSGWDFYEEEDS------KKKEQERKEKK-----------
```

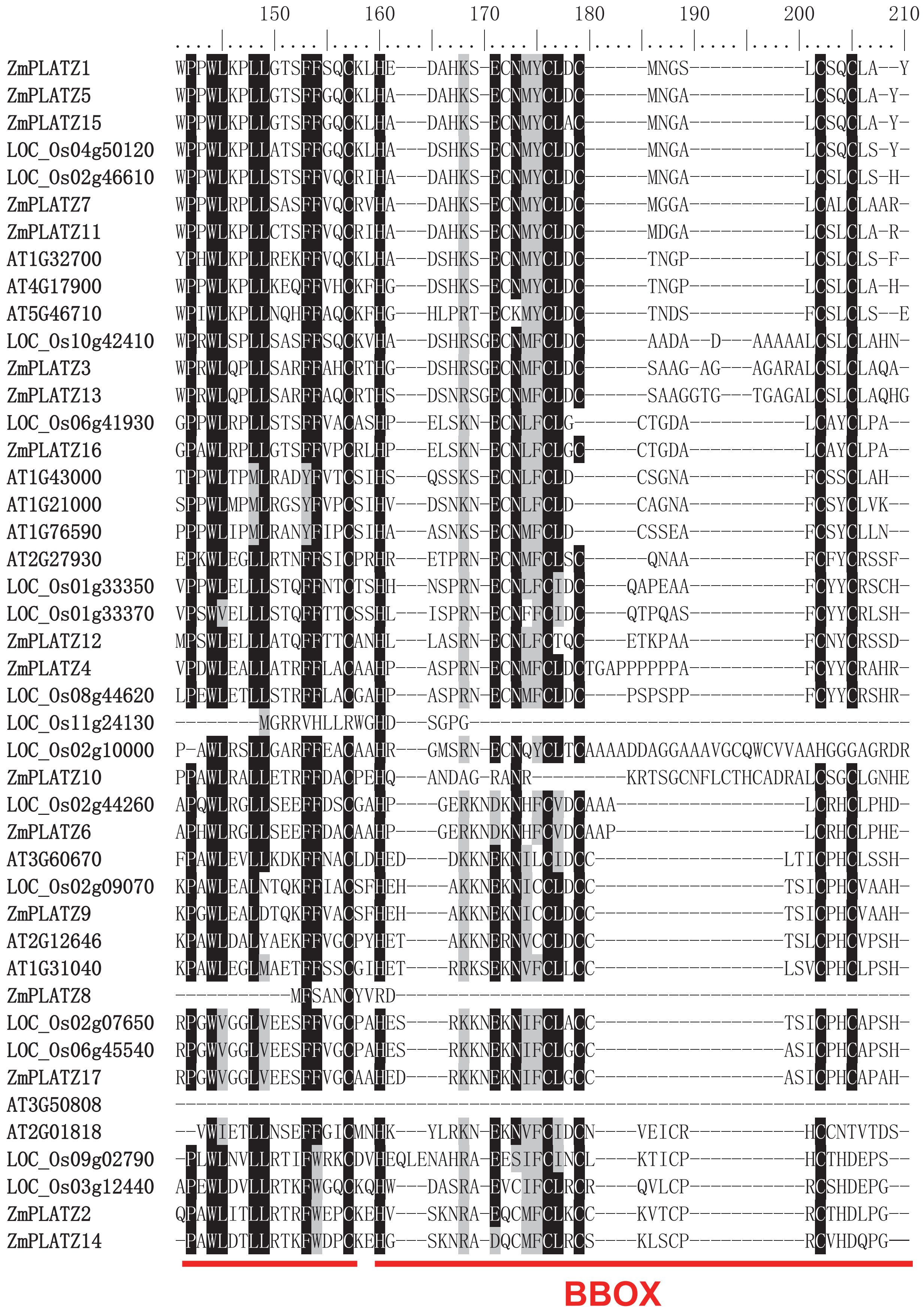
150
160
170
180
190
200
210
ZmPLATZ1
ZmPLATZ5
ZmPLATZ15
LOC_Os04g50120
LOC_Os02g46610
ZmPLATZ7
ZmPLATZ11
AT1G32700
AT4G17900
AT5G46710
LOC_Os10g42410
ZmPLATZ3
ZmPLATZ13
LOC_Os06g41930
ZmPLATZ16
AT1G43000
AT1G21000
AT1G76590
AT2G27930
LOC_Os01g33350
LOC_Os01g33370
ZmPLATZ12
ZmPLATZ4
LOC_Os08g44620
LOC_Os11g24130
LOC_Os02g10000
ZmPLATZ10
LOC_Os02g44260
ZmPLATZ6
AT3G60670
LOC_Os02g09070
ZmPLATZ9
AT2G12646
AT1G31040
ZmPLATZ8
LOC_Os02g07650
LOC_Os06g45540
ZmPLATZ17
AT3G50808
AT2G01818
LOC_Os09g02790
LOC_Os03g12440
ZmPLATZ2
ZmPLATZ14
BBOX

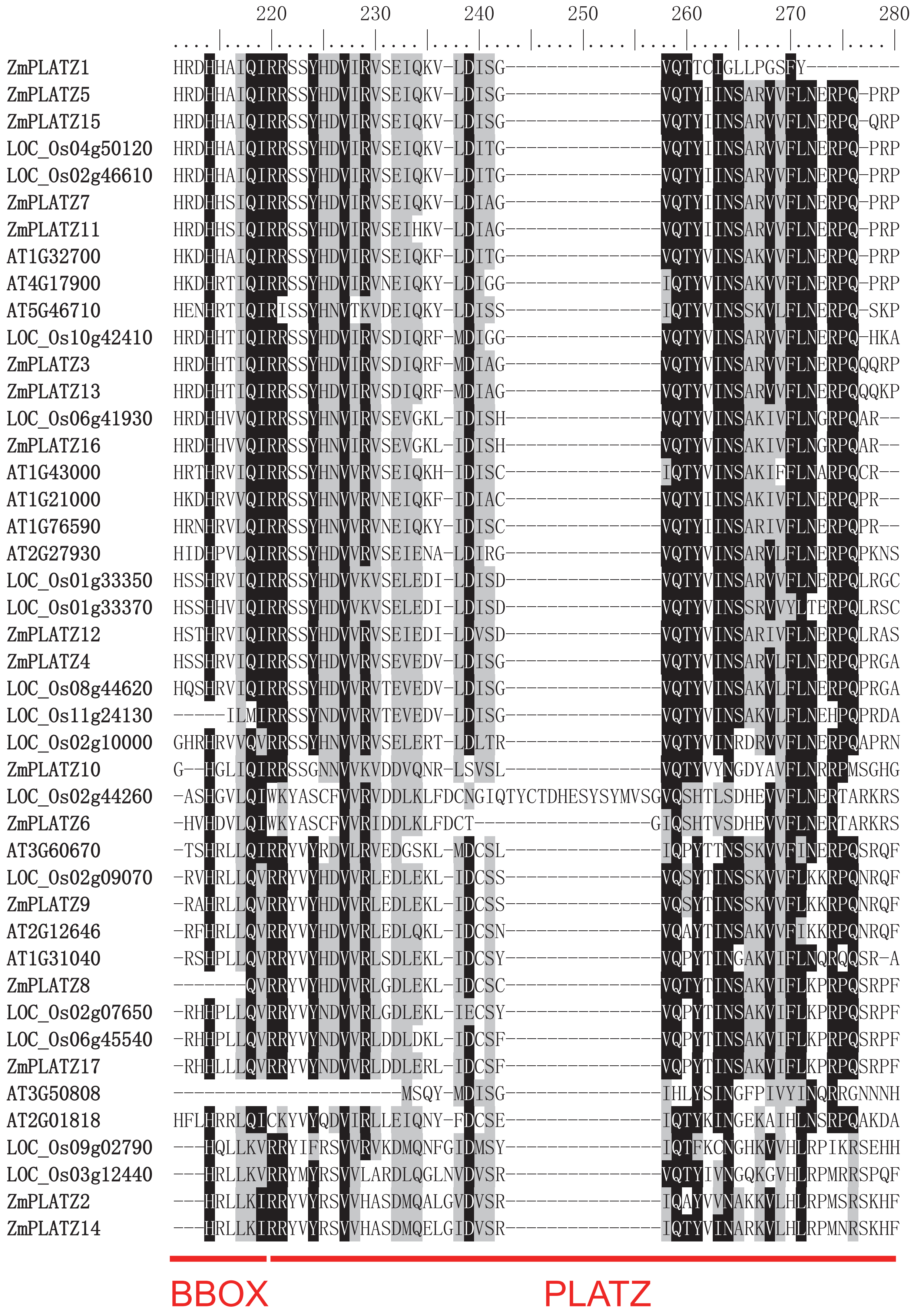
220 230 240 250 260 270 280
ZmPLATZ1 HRDHHAIQIRRSSYHDVIRVSEIQKV-LDISG---------------VQTTCIGLLPGSFY---------
ZmPLATZ5 HRDHHAIQIRRSSYHDVIRVSEIQKV-LDISG---------------VQTYIINSARVVFLNERPQ-PRP
ZmPLATZ15 HRDHHAIQIRRSSYHDVIRVSEIQKV-LDISG---------------VQTYIINSARVVFLNERPQ-QRP
LOC_Os04g50120 HRDHHAIQIRRSSYHDVIRVSEIQKV-LDITG---------------VQTYIINSARVVFLNERPQ-PRP
LOC_Os02g46610 HRDHHAIQIRRSSYHDVIRVSEIQKV-LDITG---------------VQTYIINSARVVFLNERPQ-PRP
ZmPLATZ7 HRDHHSIQIRRSSYHDVIRVSEIQKV-LDIAG---------------VQTYIINSARVVFLNERPQ-PRP
ZmPLATZ11 HRDHHSIQIRRSSYHDVIRVSEIHKV-LDIAG---------------VQTYIINSARVVFLNERPQ-PRP
AT1G32700 HKDHHAIQIRRSSYHDVIRVSEIQKF-LDITG---------------VQTYVINSAKVVFLNERPQ-PRP
AT4G17900 HKDHRTIQIRRSSYHDVIRVNEIQKY-LDIGG---------------IQTYVINSAKVVFLNERPQ-PRP
AT5G46710 HENHRTIQIRISSYHNVTKVDEIQKY-LDISS---------------IQTYVINSSKVLFLNERPQ-SKP
LOC_Os10g42410 HRDHHTIQIRRSSYHDVIRVSDIQRF-MDIGG---------------VQTYVINSARVVFLNERPQ-HKA
ZmPLATZ3 HRDHHTIQIRRSSYHDVIRVSDIQRF-MDIAG---------------VQTYVINSARVVFLNERPQQQRP
ZmPLATZ13 HRDHHTIQIRRSSYHDVIRVSDIQRF-MDIAG---------------VQTYVINSARVVFLNERPQQQKP
LOC_Os06g41930 HRDHHVVQIRRSSYHNVIRVSEVGKL-IDISH---------------VQTYVINSAKIVFLNGRPQAR--
ZmPLATZ16 HRDHHVVQIRRSSYHNVIRVSEVGKL-IDISH---------------VQTYVINSAKIVFLNGRPQAR--
AT1G43000 HRTHRVIQIRRSSYHNVVRVSEIQKH-IDISC---------------IQTYVINSAKIFFLNARPQCR--
AT1G21000 HKDHRVVQIRRSSYHNVVRVNEIQKF-IDIAC---------------VQTYIINSAKIVFLNERPQPR--
AT1G76590 HRNHRVLQIRRSSYHNVVRVNEIQKY-IDISC---------------VQTYIINSARIVFLNERPQPR--
AT2G27930 HIDHPVLQIRRSSYHDVVRVSEIENA-LDIRG---------------VQTYVINSARVLFLNERPQPKNS
LOC_Os01g33350 HSSHRVIQIRRSSYHDVVKVSELEDI-LDISD---------------VQTYVINSARVVFLNERPQLRGC
LOC_Os01g33370 HSSHHVIQIRRSSYHDVVKVSELEDI-LDISD---------------VQTYVINSSRVVYLTERPQLRSC
ZmPLATZ12 HSTHRVIQIRRSSYHDVVRVSEIEDI-LDVSD---------------VQTYVINSARIVFLNERPQLRAS
ZmPLATZ4 HSSHRVIQIRRSSYHDVVRVSEVEDV-LDISG---------------VQTYVINSARVLFLNERPQPRGA
LOC_Os08g44620 HQSHRVIQIRRSSYHDVVRVTEVEDV-LDISG---------------VQTYVINSAKVLFLNERPQPRGA
LOC_Os11g24130 ------ILMIRRSSYNDVVRVTEVEDV-LDISG---------------VQTYVINSAKVLFLNEHPQPRDA
LOC_Os02g10000 GHRHRVVQVRRSSYHNVVRVSELERT-LDLTR---------------VQTYVINRDRVVFLNERPQAPRN
ZmPLATZ10 G--HGLIQIRRSSGNNVVKVDDVQNR-LSVSL---------------VQTYVYNGDYAVFLNRRPMSGHG
LOC_Os02g44260 -ASHGVLQIWKYASCFVVRVDDLKLFDCNGIQTYCTDHESYSYMVSGVQSHTLSDHEVVFLNERTARKRS
ZmPLATZ6 -HVHDVLQIWKYASCFVVRIDDLKLFDCT-----------------GIQSHTVSDHEVVFLNERTARKRS
AT3G60670 -TSHRLLQIRRYVYRDVLRVEDGSKL-MDCSL---------------IQPYTTNSSKVVFINERPQSRQF
LOC_Os02g09070 -RVHRLLQVRRYVYHDVVRLEDLEKL-IDCSS---------------VQSYTINSSKVVFLKKRPQNRQF
ZmPLATZ9 -RAHRLLQVRRYVYHDVVRLEDLEKL-IDCSS---------------VQSYTINSSKVVFLKKRPQNRQF
AT2G12646 -RFHRLLQVRRYVYHDVVRLEDLQKL-IDCSN---------------VQAYTINSAKVVFIKKRPQNRQF
AT1G31040 -RSHPLLQVRRYVYHDVVRLSDLEKL-IDCSY---------------VQPYTINGAKVIFLNQRQQSR-A
ZmPLATZ8 -------QVRRYVYHDVVRLGDLEKL-IDCSC---------------VQTYTINSAKVIFLKPRPQSRPF
LOC_Os02g07650 -RHHPLLQVRRYVYNDVVRLGDLEKL-IECSY---------------VQPYTINSAKVIFLKPRPQSRPF
LOC_Os06g45540 -RHHPLLQVRRYVYNDVVRLDDLDKL-IDCSF---------------VQPYTINSAKVIFLKPRPQSRPF
ZmPLATZ17 -RHHLLLQVRRYVYNDVVRLDDLERL-IDCSF---------------VQPYTINSAKVIFLKPRPQSRPF
AT3G50808 ----------------------MSQY-MDISG---------------IHLYSINGFPIVYINQRRGNNNH
AT2G01818 HFLHRRLQICKYVYQDVIRLLEIQNY-FDCSE---------------IQTYKINGEKAIHLNSRPQAKDA
LOC_Os09g02790 ---HQLLKVRRYIFRSVVRVKDMQNFGIDMSY---------------IQTFKCNGHKVVHLRPIKRSEHH
LOC_Os03g12440 ---HRLLKVRRYMYRSVVLARDLQGLNVDVSR---------------VQTYIVNGQKGVHLRPMRRSPQF
ZmPLATZ2 ---HRLLKIRRYVYRSVVHASDMQALGVDVSR---------------IQAYVVNAKKVLHLRPMSRSKHF
ZmPLATZ14 ---HRLLKIRRYVYRSVVHASDMQELGIDVSR---------------IQTYVINARKVLHLRPMNRSKHF
BBOX
PLATZ

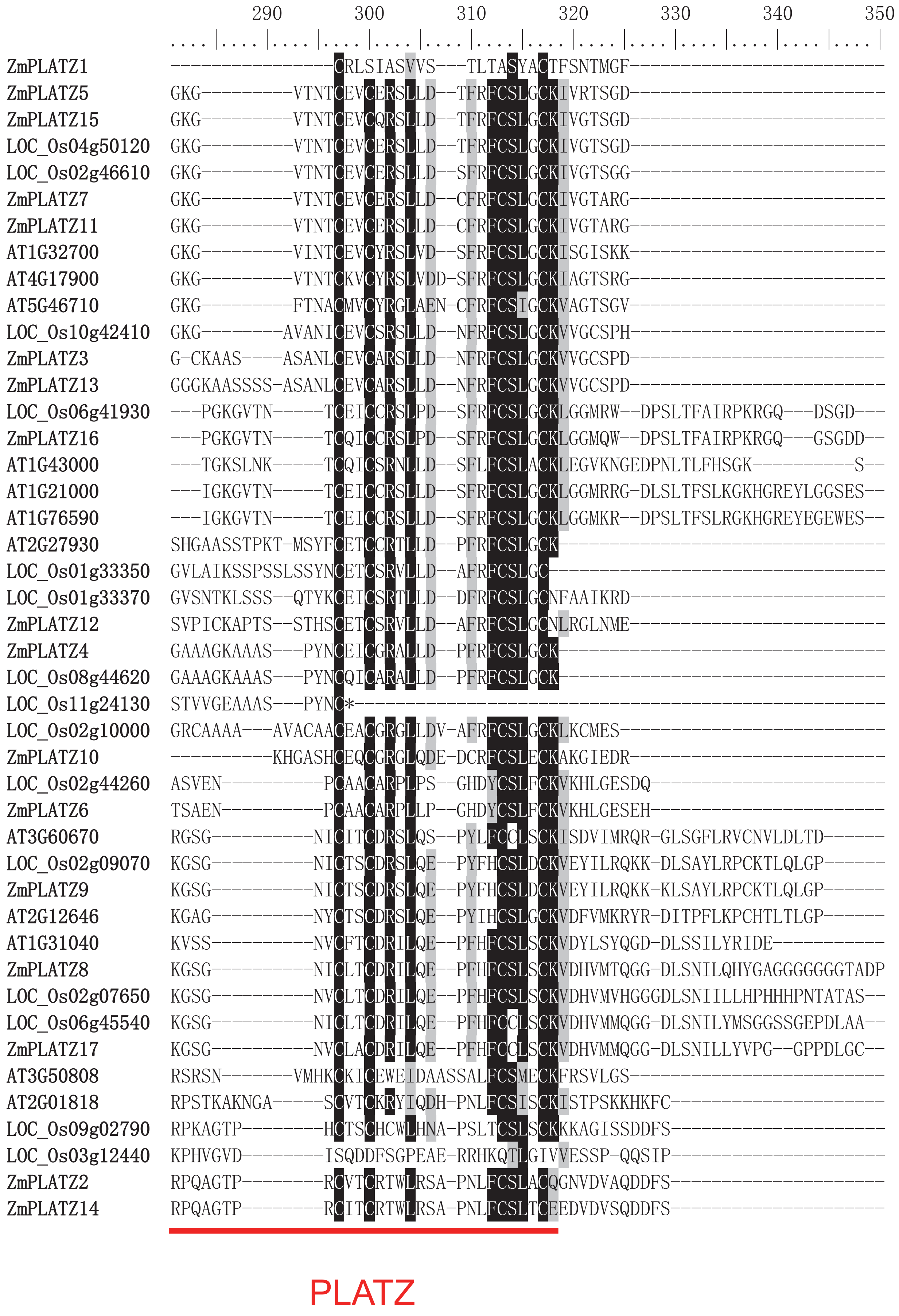
290 300 310 320 330 340 350
ZmPLATZ1 ----------------CRLSIASVVS---TLTASYACTFSNTMGF-------------------------
ZmPLATZ5 GKG---------VTNTCEVCERSLLD--TFRFCSLGCKIVRTSGD-------------------------
ZmPLATZ15 GKG---------VTNTCEVCQRSLLD--TFRFCSLGCKIVGTSGD-------------------------
LOC_Os04g50120 GKG---------VTNTCEVCERSLLD--TFRFCSLGCKIVGTSGD-------------------------
LOC_Os02g46610 GKG---------VTNTCEVCERSLLD--SFRFCSLGCKIVGTSGG-------------------------
ZmPLATZ7 GKG---------VTNTCEVCERSLLD--CFRFCSLGCKIVGTARG-------------------------
ZmPLATZ11 GKG---------VTNTCEVCERSLLD--CFRFCSLGCKIVGTARG-------------------------
AT1G32700 GKG---------VINTCEVCYRSLVD--SFRFCSLGCKISGISKK-------------------------
AT4G17900 GKG---------VTNTCKVCYRSLVDD-SFRFCSLGCKIAGTSRG-------------------------
AT5G46710 GKG---------FTNACMVCYRGLAEN-CFRFCSIGCKVAGTSGV-------------------------
LOC_Os10g42410 GKG--------AVANICEVCSRSLLD--NFRFCSLGCKVVGCSPH-------------------------
ZmPLATZ3 G-CKAAS----ASANLCEVCARSLLD--NFRFCSLGCKVVGCSPD-------------------------
ZmPLATZ13 GGGKAASSSS-ASANLCEVCARSLLD--NFRFCSLGCKVVGCSPD-------------------------
LOC_Os06g41930 ---PGKGVTN-----TCEICCRSLPD--SFRFCSLGCKLGGMRW--DPSLTFAIRPKRGQ---DSGD---
ZmPLATZ16 ---PGKGVTN-----TCQICCRSLPD--SFRFCSLGCKLGGMQW--DPSLTFAIRPKRGQ---GSGDD--
AT1G43000 ---TGKSLNK-----TCQICSRNLLD--SFLFCSLACKLEGVKNGEDPNLTLFHSGK----------S--
AT1G21000 ---IGKGVTN-----TCEICCRSLLD--SFRFCSLGCKLGGMRRG-DLSLTFSLKGKHGREYLGGSES--
AT1G76590 ---IGKGVTN-----TCEICCRSLLD--SFRFCSLGCKLGGMKR--DPSLTFSLRGKHGREYEGEWES--
AT2G27930 SHGAASSTPKT-MSYFCETCCRTLLD--PFRFCSLGCK--------------------------------
LOC_Os01g33350 GVLAIKSSPSSLSSYNCETCSRVLLD--AFRFCSLGC---------------------------------
LOC_Os01g33370 GVSNTKLSSS--QTYKCEICSRTLLD--DFRFCSLGCNFAAIKRD-------------------------
ZmPLATZ12 SVPICKAPTS--STHSCETCSRVLLD--AFRFCSLGCNLRGLNME-------------------------
ZmPLATZ4 GAAAGKAAAS---PYNCEICGRALLD--PFRFCSLGCK--------------------------------
LOC_Os08g44620 GAAAGKAAAS---PYNCQICARALLD--PFRFCSLGCK--------------------------------
LOC_Os11g24130 STVVGEAAAS---PYNC*---
LOC_Os02g10000 GRCAAAA---AVACAACEACGRGLLDV-AFRFCSLGCKLKCMES--------------------------
ZmPLATZ10 ----------KHGASHCEQCGRGLQDE-DCRFCSLECKAKGIEDR-------------------------
LOC_Os02g44260 ASVEN----------PCAACARPLPS--GHDYCSLFCKVKHLGESDQ-----------------------
ZmPLATZ6 TSAEN----------PCAACARPLLP--GHDYCSLFCKVKHLGESEH-----------------------
AT3G60670 RGSG----------NICITCDRSLQS--PYLFCCLSCKISDVIMRQR-GLSGFLRVCNVLDLTD------
LOC_Os02g09070 KGSG----------NICTSCDRSLQE--PYFHCSLDCKVEYILRQKK-DLSAYLRPCKTLQLGP------
ZmPLATZ9 KGSG----------NICTSCDRSLQE--PYFHCSLDCKVEYILRQKK-KLSAYLRPCKTLQLGP------
AT2G12646 KGAG----------NYCTSCDRSLQE--PYIHCSLGCKVDFVMKRYR-DITPFLKPCHTLTLGP------
AT1G31040 KVSS----------NVCFTCDRILQE--PFHFCSLSCKVDYLSYQGD-DLSSILYRIDE-----------
ZmPLATZ8 KGSG----------NICLTCDRILQE--PFHFCSLSCKVDHVMTQGG-DLSNILQHYGAGGGGGGGTADP
LOC_Os02g07650 KGSG----------NVCLTCDRILQE--PFHFCSLSCKVDHVMVHGGGDLSNIILLHPHHHPNTATAS--
LOC_Os06g45540 KGSG----------NICLTCDRILQE--PFHFCCLSCKVDHVMMQGG-DLSNILYMSGGSSGEPDLAA--
ZmPLATZ17 KGSG----------NVCLACDRILQE--PFHFCCLSCKVDHVMMQGG-DLSNILLYVPG--GPPDLGC--
AT3G50808 RSRSN-------VMHKCKICEWEIDAASSALFCSMECKFRSVLGS-------------------------
AT2G01818 RPSTKAKNGA-----SCVTCKRYIQDH-PNLFCSISCKISTPSKKHKFC---------------------
LOC_Os09g02790 RPKAGTP--------HCTSCHCWLHNA-PSLTCSLSCKKKAGISSDDFS---------------------
LOC_Os03g12440 KPHVGVD--------ISQDDFSGPEAE-RRHKQTLGIVVESSP-QQSIP---------------------
ZmPLATZ2 RPQAGTP--------RCVTCRTWLRSA-PNLFCSLACQGNVDVAQDDFS---------------------
ZmPLATZ14 RPQAGTP--------RCITCRTWLRSA-PNLFCSLTCEEDVDVSQDDFS---------------------
PLATZ

```
                        360       370       380       390       400       410       420
                ....|....|....|....|....|....|....|....|....|....|....|....|....|....|
ZmPLATZ1        -----------------------*----------------------------------------------
ZmPLATZ5        -----------------------FRIRKKHAIVAKKKREKKHAP----------QQKQHRGAADSADDDD
ZmPLATZ15       -----------------------LRIRKKQAVVKKHQKKKKQ------------QAQQHRGAA--LDSED
LOC_Os04g50120  -----------------------YRGRKRHAGGGIKKT-----------------KKLHKGAA-AVPSDS
LOC_Os02g46610  -----------------------YRPRKKHGGCGGGGGGGDGG-----------KKKKKRAALKDARYES
ZmPLATZ7        -----------------------YRPGKKKHG-GGGGNK---------------KRASPAPALKDVRSDS
ZmPLATZ11       -----------------------YRP-KKKHGSGGGGGN---------------KRKR--AALKDVRSDS
AT1G32700       -----------------------KRKEWTNNL---------------------------------SDSDD
AT4G17900       -----------------------FEKGRENLL---------------------------------METED
AT5G46710       -----------------------FQKRVKHTTNDSD------------------------------NSNN
LOC_Os10g42410  -----------------------AATAAATATATAARRK-------------------------RLRHAH
ZmPLATZ3        -----------------------AAKARSWLLRPAVGG------------------------------SG
ZmPLATZ13       -----------------------AAKARNWLLRAAD---------------------------------G
LOC_Os06g41930  ---------------GGSGSDYDSFSPKKARRAAAGYDQ-------------------------LGRFDR
ZmPLATZ16       ---------------GGSGSD-DSFSPKKPRR-MAGFD--------------------------LGRFER
AT1G43000       ---------------DDS---SKIINTGICSRLIDGIS--------------------------IAVD--
AT1G21000       ---------------DEATTPTKMRKTNAFNRLMSGLS--------------------------ISTVRF
AT1G76590       ---------------DEATTPTKIRKTCAFNRLMSGLS--------------------------ISTVKC
AT2G27930       ------------------------VEGMRKNKEEEEER--------------------------LRKER-
LOC_Os01g33350  -----------------------NVSYKPLQQVLFKLI--------------------------LLCN--
LOC_Os01g33370  ------------------N--EKNVAQNGIASNANEVK--------------------------IGTNNG
ZmPLATZ12       ------------------AGMQAMVGNNPRSNGMDHVA--------------------------RIDNVG
ZmPLATZ4        -----------------------LVDTKRSNGHAASSA--------------------------DGGGGG
LOC_Os08g44620  -----------------------LVDTK-TGGRGATVQ--------------------------PGD---
LOC_Os11g24130  ----------------------------------------------------------------------
LOC_Os02g10000  ------------------------DPTLTFTIDPNNIPEPQIS--------------GPQEDEEEDDDEE
ZmPLATZ10       -----------------------LDFSVSFAVDPNNFS-------------------SSGDDTESDDDED
LOC_Os02g44260  ----------------------GLRRALRVNRRSAAAAG-------------------------------
ZmPLATZ6        ----------------------ELRRALRVSRKEVAPT--------------------------------
AT3G60670       ---EVTTTTPSSTLEPT-----GSNRTSSESSGNEGE-----------DMFWCQALACT-----------
LOC_Os02g09070  ---DFFIPHD------------ADDETTHSTLVDVDE-----------PMG--SSDSEN--------LSA
ZmPLATZ9        ---DFFIPHD------------ADDDTTHSTLVDVDE-----------PMG--SSDSEN--------LSV
AT2G12646       ---DYIIPQDLLTDDEV-----AAYETPRSTVVDGDE-----------SMSWSSASSDNNNAGAAAAYAA
AT1G31040       ---SDFTFEGLRMDG--------HDQLGEISTMEDGE-----------DILVISDESEQGNN------SH
ZmPLATZ8        DRLAFPRFENLRVVD-------GSDLDDDVQVVTPDSTLEDP------TNNAGGGSSDNGTD------DA
LOC_Os02g07650  ---AFPRFEDLRVG--------ADDDAAAITAVTPE----------GRYGGGGGGSSDNGGG------DG
LOC_Os06g45540  ---GFPRFENLRVDGGGGGGGGLSDDDDDHQVTTPNSILEDPLHHHHHQYYGGGGSSNNGRS------TS
ZmPLATZ17       ---GFPRFENLRFD----------DDPGQYGQATPSSILEDPVEHGGSASASGG-SSNGGSA------RN
AT3G50808       ----------------------QLDELMENSSEVTEIS--------------------------------
AT2G01818       ----------------------FSPKLEQSVLEKEHSTQEGS-------------------------LEE
LOC_Os09g02790  ----------------------GPEASTRVSRSRNHASNVN-----------------------------
LOC_Os03g12440  ----------------------QPFDASPVRNEDATMVEAEC-------------------------GQV
ZmPLATZ2        ----------------------GPEAEVRYRSLQVQMAEPSG-------------------------AAA
ZmPLATZ14       ----------------------GPEAELRYRSFQVHMAEP----------------------------AE
```

```
                        430       440       450       460       470       480       490
               ....|....|....|....|....|....|....|....|....|....|....|....|....|....|
ZmPLATZ1       ----------------------------------------------------------------------
ZmPLATZ5       DDSSTSTSGGS-DKSSVVQSFTPSTPPATTANSFRAG-KRRKGVPHRSPFGSLVVEF*------------
ZmPLATZ15      DSSSTSTSRGS-DRSSVVQSFTPSTPPAT-ANSFRTG-KRRKGVPHRSPFGSLMVEEF*-----------
LOC_Os04g50120 DDSSTTTSGGS-DKSSVVQSFTPSTPPAT-ANSYRTG-KRRKGVPHRSPFGSLMVEF*------------
LOC_Os02g46610 EDSCTSTSGGSSDKSSVVQSFTPLTPPPTSASYR-TGNKRRKGVPHRSPFGSLIVEF*------------
ZmPLATZ7       EDSCTSTSGASSDKSSVVQS---SPPPPTSASHRPPGNKRRKGIPHRSPLGSLIVEL*------------
ZmPLATZ11      EESCTSTSGASSDKSSVVQSFSPSTPPPASASYRRPGNKRRKGVPHRSPFGSLIVEF*------------
AT1G32700      SYSSTSIGRLKKNDDIMNNSFTPSTPPLS-AVNRRIA-KRRKGIPHRAPFGGLIIEY*------------
AT4G17900      SSSSIAIGKNITN----LQSFSPSTPPLTTSSNCRIV-KRRKGIPHRSPMG*------------------
AT5G46710      SSGVENNSSGAENGNSNLQSLSPPTPQFPPRS---LRKRLRKGIPHRAPFS*------------------
LOC_Os10g42410 AMASTSDSDNS--TSPAKRSFTPSTPPPPPT----LPPKRRKGIPHRAPFGSLIVEY*------------
ZmPLATZ3       DGDSTSSSPLR--DAQKRQSFTP--PTPQP-------AKRRKGIPHRAPFGSFIVEY*------------
ZmPLATZ13      DGSTTSSSAPR--NADRKLSFTP--PTPQPT----LPTKRRKGIPHRAPFGSLIVEY*------------
LOC_Os06g41930 -GMIRWSDDEGSKSNTA---PITPTTPPISRCRPS----RRKGIPHRAPFYG*-----------------
ZmPLATZ16      PGGIRWSDDEGSRSNNG---LITPGTPPINRCRPS----RRKGIPHRAPFYG*-----------------
AT1G43000      -------DQRSETAG-----VLSPETPSIESHRNYPMKSRRKGIPQRAPF*-------------------
AT1G21000      DDYGPNGDQRSSSSGDEGGFSFSPGTPPIYNHRNS---SRRKGVPHRAPF*-------------------
AT1G76590      DYLS--GDQPSSSSGDESGFKLSPGTPPIYNHRNS---SRRKGVPHRAPF*-------------------
AT2G27930      ------------QQETH-----KGTHPPTHTSNSR----RRKGIPHRAPFAS------------------
LOC_Os01g33350 -------------------------------LICS----QILG*--------------------------
LOC_Os01g33370 STNAGSANEISSDANNY-----RNEIPSSTRVIRH----RRKGIPRRAPFF*------------------
ZmPLATZ12      SSTTNDQN----SCNDK-----NYEEPPPKRVARH----RRKGIPQRAPFF*------------------
ZmPLATZ4       GGAASGNDE---TTEAG-----GSKNGPGARPHGR----RRKGTPHRAPFWS*-----------------
LOC_Os08g44620 ---ATNDDA---AAAGG-----SSKNG-GARPQGR----RRKGIPQRAPFGS*-----------------
LOC_Os11g24130 ----------------------------------------------------------------------
LOC_Os02g10000 DEPFYPTKANAAQSKAAGGGGCRPPPPPSASSSRPRRGGRRVARGDKEEDQEAAANILAFAAAAARSVPA
ZmPLATZ10      SS--YPSKFQKLETIPAS-------------SSKPVASGGQHSIGKKQY*--------------------
LOC_Os02g44260 -GGGGGEDPAVAEASQSGKRRASSSESGRSCGGTLRKRSRKQPAPARSPSC*------------------
ZmPLATZ6       -----------PEPQTGRKRSSSSSDAGPSCGGSFRKRSRKQAEPAQAPFH*------------------
AT3G60670      ATTEIVRKKRSSLSTTCRRVT--EVVSTTNTEAPVNFLNRRKNPPQRAPLY*------------------
LOC_Os02g09070 PCPNFVRKKRSGPYICARSAN---RVSDD--DMATN-MSRRKGVPHRSPLC-------------------
ZmPLATZ9       PCTNFVRKKRSGPYICARSAN---RVSEE--DMATN-MSRRKGVPQRSPLC*------------------
AT2G12646      TTTHVVRKKRTGFCLCAKSANSYKEVSEDPDDISAC-INRRKGVPQRSPLC*------------------
AT1G31040      KKE-----KKKSKKKKPESN-YLPGMVLSSLG------NRRKGAPHRAPFS*------------------
ZmPLATZ8       RRQVVVHGGGEAAKRKKGGG-FLPQIVLS-LGGGGGGGNRRKGAPHRSPLA*------------------
LOC_Os02g07650 GGGEVGEVKRKKKK---GGG-FFPQILG--LG------SRRKGAPHRSPLS*------------------
LOC_Os06g45540 PAPTTADVP-RKKKSGGGGG-FFPQIVLS-LN------NRRKGAPHRSPLA*------------------
ZmPLATZ17      HARRDVDVPTRKKKSGGGGGGFFPQIVLS-LG------NRRKGAPHRAPLA*------------------
AT3G50808      ---------------------------EEIDEPVMKKRHRRKGSPHRAPFF-------------------
AT2G01818      KKSCTSSLTDVSEDSEVLLS--------DFSFRPLLRILKRKGISRRSPFY*------------------
LOC_Os09g02790 ----------------QKHP----------TNTKLRKKPRKQANPERAPFF*------------------
LOC_Os03g12440 QTNATESESSAVGDADEVIPKVTKFN---VDIHSLRRRVRKQAAPQRAPFF*------------------
ZmPLATZ2       DELPG-PEAAHEV-PAQVPPPPPP---AANQNASLRRRPRKQAAPERAPFF*------------------
ZmPLATZ14      ELLPDDPEVEHEIMPAQVEPPPPAAAAAANQNVSLRRRARKQAAPLRAPFF*------------------
```

```
                     500       510
               ....|....|....|....|....|...
ZmPLATZ1       ----------------------------
ZmPLATZ5       ----------------------------
ZmPLATZ15      ----------------------------
LOC_Os04g50120 ----------------------------
LOC_Os02g46610 ----------------------------
ZmPLATZ7       ----------------------------
ZmPLATZ11      ----------------------------
AT1G32700      ----------------------------
AT4G17900      ----------------------------
AT5G46710      ----------------------------
LOC_Os10g42410 ----------------------------
ZmPLATZ3       ----------------------------
ZmPLATZ13      ----------------------------
LOC_Os06g41930 ----------------------------
ZmPLATZ16      ----------------------------
AT1G43000      ----------------------------
AT1G21000      ----------------------------
AT1G76590      ----------------------------
AT2G27930      ----------------------------
LOC_Os01g33350 ----------------------------
LOC_Os01g33370 ----------------------------
ZmPLATZ12      ----------------------------
ZmPLATZ4       ----------------------------
LOC_Os08g44620 ----------------------------
LOC_Os11g24130 ----------------------------
LOC_Os02g10000 ASAADPNSYRRRARKGAHRAPERSPFF*
ZmPLATZ10      ----------------------------
LOC_Os02g44260 ----------------------------
ZmPLATZ6       ----------------------------
AT3G60670      ----------------------------
LOC_Os02g09070 ----------------------------
ZmPLATZ9       ----------------------------
AT2G12646      ----------------------------
AT1G31040      ----------------------------
ZmPLATZ8       ----------------------------
LOC_Os02g07650 ----------------------------
LOC_Os06g45540 ----------------------------
ZmPLATZ17      ----------------------------
AT3G50808      ----------------------------
AT2G01818      ----------------------------
LOC_Os09g02790 ----------------------------
LOC_Os03g12440 ----------------------------
ZmPLATZ2       ----------------------------
ZmPLATZ14      ----------------------------
```

Supplemental Fig4 Amino Acid Sequence Alignment of ZmPLATZs, AtPLATZs and OsPLATZs. Black shaded amino acids represent identical amino acid residues and gray ones indicate the similar amino acid residues.
